# Neural mechanisms of the relationship between aerobic fitness and working memory in older adults: An fNIRS study

**DOI:** 10.1162/imag_a_00167

**Published:** 2024-05-10

**Authors:** Kazuki Hyodo, Ippeita Dan, Takashi Jindo, Kiyomitsu Niioka, Sho Naganawa, Ayako Mukoyama, Hideaki Soya, Takashi Arao

**Affiliations:** Physical Fitness Research Institute, Meiji Yasuda Life Foundation of Health and Welfare, Tokyo, Japan; Applied Cognitive Neuroscience Lab., Research and Development Initiatives, Chuo University, Tokyo, Japan; Special Appointed Lecturer, Division of Art, Music, and Physical Education, Osaka Kyoiku University, Osaka, Japan; Department of Physiological Science, The University of Human Environments, Ehime, Japan; Laboratory of Exercise Biochemistry and Neuroendocrinology, Faculty of Health and Sport Sciences, University of Tsukuba, Ibaraki, Japan; Sport Neuroscience Division, Advanced Research Initiative for Human High Performance (ARIHHP), Faculty of Health and Sport Sciences, University of Tsukuba, Ibaraki, Japan

**Keywords:** aerobic fitness, aging, working memory, n-back task, prefrontal cortex, functional near infrared spectroscopy

## Abstract

A growing number of studies have revealed that higher aerobic fitness is associated with better working memory (WM) performance in older adults. However, the underlying functional neural mechanisms of this association remain under debate. It has been reported that aging increases recruitment of the prefrontal cortex (PFC) during cognitive tasks, and that this is associated with task performance in a compensatory manner. Therefore, this study aimed to clarify the prefrontal activation pattern that is associated with the relationship between aerobic fitness and WM performance in older adults, focusing on age-related extended prefrontal recruitment. Forty-seven older adults (65–74 years, 29 females) and 49 younger adults (18–24 years, 23 female) performed verbal and spatial n-back tasks, which included 0-, 1-, and 2-back conditions. Reaction time (RT) and accuracy (ACC) were assessed as indices of task performance. Prefrontal activation during the experimental tasks was monitored using functional near-infrared spectroscopy (fNIRS) and analyzed using an adaptive GLM method. We compared task performance and prefrontal activation between age groups to find age-related prefrontal activation patterns. Only older adults underwent a graded exercise test (GXT) to determine their ventilation thresholds (VT) as a measure of aerobic fitness, and, subsequently, the relationships among aerobic fitness, n-back task performance, and prefrontal activation in older adults were examined using correlation analysis and mediation analysis controlling for possible covariates. A comparison of task performance between groups revealed that older adults had slower RT and lower ACC than did younger adults, especially in the higher WM load 2-back condition. Group comparisons of prefrontal activation showed that older adults exhibited additional or greater activation than younger adults mainly in the ventrolateral PFC (VLPFC) and front polar area (FPA) in both the verbal and spatial 2-back conditions. Correlation analysis showed a relationship between higher VT, shorter RT for the verbal 2-back condition, and greater prefrontal activation of the bilateral FPA and right VLPFC during verbal 2-back conditions in older adults. In addition, mediation analyses indicated the possibility of a mediation effect of the prefrontal activation on the relationship between VT and RT for the verbal 2-back condition. These results suggest that older adults with higher aerobic fitness levels recruited more extended PFC regions, possibly for compensatory activation, to enhance their performance of the verbal n-back task. This study sheds light on the neural mechanisms underpinning the relationship between aerobic fitness and cognitive function in older adults.

## 1 Introduction

Normal aging is associated with decline in several cognitive functions, such as long-term memory, processing speed, and problem solving. Among them, executive function, which refers to complex and higher-order cognitive control processes mainly associated with the prefrontal cortex (PFC), is vulnerable to aging (D. C. Park et al., 2002;Idowu & Szameitat, 2023;Kumar & Priyadarshi, 2013). Executive function has three core components: working memory (WM), inhibition, and shifting (Miyake et al., 2000). Since executive function is crucially involved in daily activities, preventing its decline with aging could help maintain a higher quality of life (QOL) in older adults.

It has been reported that there are great individual differences in the decline of executive function with aging (Ardila, 2008). Among lifestyle factors that may affect the rate and extent of such decline, physical activity has been suggested as being influential. Many studies have examined the effects of physical activity and exercise on age-related functional decline and reported their beneficial effects on executive functions (Hillman et al., 2008;Kramer & Colcombe, 2018). Recent meta-analyses of exercise intervention effects have reported that physical exercise has a positive effect on the three components of executive function (Chen et al., 2020;Hoffmann et al., 2021;Zhidong et al., 2021). This is supported by previous findings showing that exercise induces molecular, cellular, and structural changes in the brain such as increases in neurotrophic factor, synaptic plasticity, and gray and white matter volume (El-Sayes et al., 2019). Moreover, cross-sectional studies have reported the association of aerobic fitness with executive function and the underlying brain mechanisms, focusing primarily on inhibitory function (Colcombe et al., 2004;Goenarjo et al., 2021;Hyodo et al., 2016;Mekari et al., 2019;Prakash et al., 2011;Voelcker-Rehage et al., 2010;Weinstein et al., 2012). On the other hand, although several studies have reported a relationship between WM, a subcomponent of executive function, and aerobic fitness (Oberlin et al., 2016;Szabo et al., 2011;Voelcker-Rehage et al., 2010;Weinstein et al., 2012), the functional neural mechanism underlying this relationship in older adults has yet to be elucidated.

To clarify this, understanding age-related changes in brain activation patterns during WM tasks is of great importance. Functional neuroimaging studies have shown that WM involves several brain regions, primarily the PFC, as well as the posterior parietal cortex, insula, and cingulate cortex (Carlson et al., 1998;Nagel et al., 2011;Owen et al., 2005;Yaple et al., 2019). WM mainly consists of two subprocesses: the phonological loop, which processes verbal information, and the visuospatial sketchpad, which processes spatial and visual information (Baddeley, 2012), and brain activation patterns have been reported as being different between verbal and spatial WM tasks: the left PFC is predominantly activated during verbal WM tasks, whereas the right PFC is predominantly or bilaterally activated during spatial WM tasks (Cona & Scarpazza, 2019;Owen et al., 2005;Thomason et al., 2009).

Studies examining age-related changes in task-related brain activation have, interestingly, often reported that older adults recruit greater or more extensive brain activation in the bilateral cerebral hemispheres, especially the PFC; this is known as overactivation. Some have theorized that this overactivation represents a dedifferentiation or decrease in neural efficiency (Dennis & Cabeza, 2011;J. Park et al., 2010). However, many studies have found a positive association between overactivation and task performance, suggesting that overactivation works in a compensatory manner, and this has been explained by several models (Festini et al., 2018;Reuter-Lorenz et al., 2021). For example, the Hemispheric Asymmetry Reduction in Older Adults (HAROLD) model (Cabeza, 2002;Reuter-Lorenz et al., 2000) represents a phenomenon in which older adults recruit bilateral PFC regions during cognitive tasks in which younger adults exhibit left- or right-dominant prefrontal activation. The Posterior-Anterior Shift in Aging (PASA) model represents the phenomenon of decreased activation of posterior regions and increased activation of anterior frontal regions in older adults. The Compensation-Related Utilization of Neural Circuits Hypothesis (CRUNCH) (Mattay et al., 2006;Reuter-Lorenz & Cappell, 2008) suggests that older adults tend to exhibit more brain activation than younger adults, but when task demands increase and reach the limit of one’s ability to compensate, overactivation no longer occurs. Regarding compensatory functions in older adults, previous neuroimaging studies have found that older adults recruit additional prefrontal areas, where younger adults do not, during WM tasks: the additional prefrontal activation is associated with better working memory performance (Sala-Llonch et al., 2012;Saliasi et al., 2014). It is highly likely that such age-related additional activation in task-related brain regions could reflect compensatory functions.

Recently, using fNIRS, which is suitable for assessing WM function because it enables hemodynamic measurement in a naturalistic environment (seeSection 2.5.1in the Methods),Agbangla et al. (2019)reported that high-fitness older adults had both greater accuracy on verbal WM tasks at higher task-demand conditions and more bilateral prefrontal activation compared to low-fitness older adults. These findings suggest that higher-fitness older adults can achieve better WM performance through more compensatory brain activation during WM-related tasks. However, previous studies examining the relationship between aerobic fitness and brain activation during inhibitory tasks have reported that older adults with higher aerobic fitness exhibited more youth-like activation patterns (Colcombe et al., 2004;Hyodo et al., 2016;Voelcker-Rehage et al., 2010). Therefore, conclusions about which prefrontal activation (task-specific youth-like activation or age-related overactivation) is associated with aerobic fitness and WM performance in older adults cannot be made.

Hence, this study tackled this issue by using verbal and spatial n-back tasks with fNIRS neuroimaging. Since prefrontal activation patterns have been reported to be different between verbal and spatial WM tasks (Cona & Scarpazza, 2019;Owen et al., 2005;Thomason et al., 2009), we posited that the use of two different tasks would more comprehensively reveal the prefrontal activation patterns underlying aerobic fitness and WM task performance in older adults. First, we compared prefrontal activation during n-back tasks between younger and older adults. In particular, we examined whether the laterality for the verbal WM task expected for younger adults disappears and/or additional cortical regions are recruited in older adults. Next, we identified n-back task performance associated with aerobic exercise capacity and clarified prefrontal activation associated with both in older adults. Taken together, these results shed light on the neural basis of the relationship between aerobic fitness and WM performance in older adults.

## 2 Methods

### 2.1 Participants

Sixty-one older adults and 51 younger adults were initially recruited for the study through advertisements in local magazines and through on-campus e-mail advertisements at Chuo University, respectively. The inclusion criteria were as follows: (a) age range of 65 to 74 years for older adults and 18 to 24 years for younger adults, (b) right-handedness, (c) normal or corrected-to-normal vision, (d) no neuropsychiatric conditions, neurological diseases, or infarcts, and (e) no physical disabilities that could be exacerbated by exercise. Demographic and clinical information was obtained from self-report questionnaires. Through the screening questionnaires and experimental process, 14 older adults and 2 younger adults were excluded for the following reasons: smoking (1 older adult), taking sleep medicine (1 older adult), taking medicine that could affect the central nervous system (4 older adults), history of brain infarct (1 older adult), possible depression (scores >7 on the Geriatric Depression Scale (GDS) in 2 older adults, and scores >12 on the Kessler Psychological Distress Scale (K6) in 2 younger adults), technical difficulty with cycling exercise (1 older adult), and below-chance performance for accuracy during n-back tasks (less than 50 %, 4 older adults). Thus, data from 47 older and 49 younger participants were used for analysis. Participant baseline characteristics are shown inTable 1. Written informed consent was obtained from every participant prior to the experiment. This study was approved by the Ethics Committee of the Physical Fitness Research Institute of the Meiji Yasuda Life Foundation of Health and Welfare and Chuo University, and the protocol was in accordance with the Declaration of Helsinki guidelines. To determine the required sample size, we conducted a power analysis for correlation using the G*Power application. Based on our previous study (Hyodo et al., 2016), the effect size (r) was set to 0.4. With an alpha level of 0.05 and a power of 0.8, the minimum estimated sample size required was 44 participants.

**Table 1.** Participant characteristics.

	Older adults (n = 47)				Younger adults (n = 49)			
	Female (n = 29)		Male (n = 18)		Female (n = 23)		Male (n = 26)	
	Mean	SD	Mean	SD	Mean	SD	Mean	SD
Age [y]	69	3.0	70	2.2	22	1.2	22	1.3
Height [cm]	153	5.8	165	6.0	158	5.4	173	5.9
Weight [kg]	50	7.0	61	7.0	52	8.8	61	8.8
Education [y]	13	1.8	14	2.2	15	1.0	15	1.4
GDS [score]	1.5	1.4	1.3	1.4				
K6 [score]					4.9	3.5	4.2	3.1
VT [ml/kg/min]	12	1.9	13	3.0				

GDS: Geriatric Depression Scale; K6: Kessler Psychological Distress Scale; VT: ventilatory threshold.

### 2.2 Procedure

The older adults underwent the experiment at the laboratory of the Physical Fitness Research Institute in Tokyo, and the younger adults underwent it at a laboratory at Chuo University in Tokyo. After arriving at the laboratory, participants first answered questionnaires about demographic and clinical data, and then performed verbal and spatial n-back tasks. Only the older adults performed a graded exercise test (GXT) after the n-back tasks. The younger adults did not perform a GXT because they were recruited as a control group to confirm age-specific prefrontal activation patterns during WM tasks. The experiments were conducted between January 2017 and May 2018.

### 2.3 Graded exercise test

To determine ventilation threshold (VT), the older adults underwent a GXT using an upright cycle ergometer (Corival cpet, Lode, Netherlands). The GXT began with a 3-min warm-up at 0 watts (W). After that, the load was increased by 1 W every 6 s. Participants were instructed to maintain a pedaling speed of 55 rotations per minute (rpm) by pedaling in time with a metronome set to 55 beats per minute (bpm). If the pedaling rate was slow (<50 rpm) or fast (>60 rpm), participants were asked to adjust their pedaling speed to match the metronome. Heart rate (HR) and rate of perceived exertion (RPE) (Borg, 1970) were recorded every minute. In order for participants to reach their VT safely, the maximal exercise load was set to correspond with an RPE of 17 (very hard). After reaching their maximum exercise load, the participants continued pedaling for a 1-min cool down while the load was gradually decreased. Oxygen intake (V̇o_2_), and carbon dioxide output (V̇co_2_) were measured breath-by-breath using an aeromonitor (Aeromonitor AE280S, Minato Medical Science, Osaka, Japan) and the respective values were averaged every 10 s, based onHarms et al. (1998). VT was determined with V-slope methods using the aeromonitor, in accordance withItoh et al. (2005). Briefly, respiratory gas measures were graphically plotted against workload, and VT was determined as the point at which the relationship between V_CO2_and workload changed from linear to non-linear.

### 2.4 N-back tasks

**Fig. 1.**
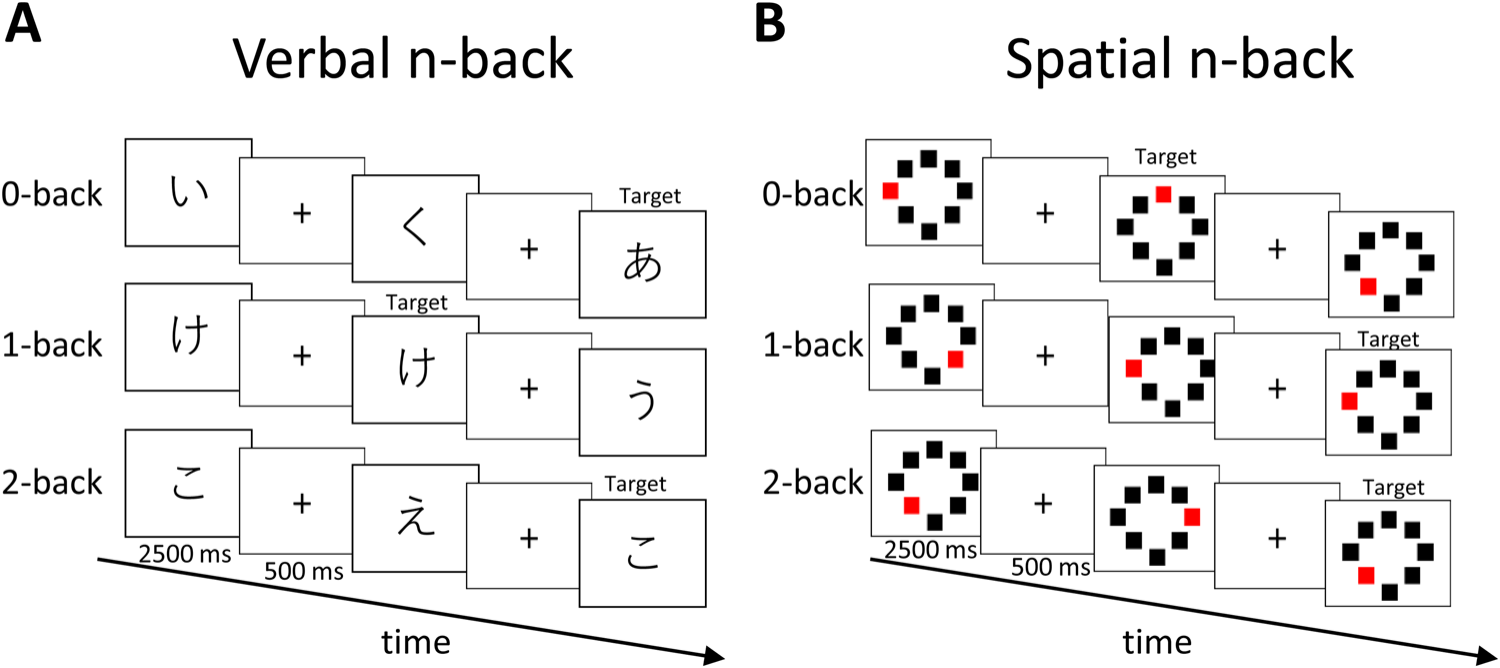
Schematic representations of the two n-back tasks. (A) Verbal n-back task. The stimulus was presented for 2,500 ms, and the inter-stimulus interval with a fixation cross was presented for 500 ms. Target stimulus for the 0-back condition was “あ.” (B) Spatial n-back task. The timing was the same as for (A). Target stimulus for the 0-back condition was the red square at the top of the circle.

Participants performed computer-based verbal and spatial n-back tasks in a random order (Fig. 1). The n-back tasks consisted of three conditions: 0-back (no WM load), 1-back (low WM load), and 2-back (high WM load). For each task, the conditions were presented in ascending order and each condition consisted of 2 blocks with 20 trials, 5 of which were target trials. Each stimulus was presented for 2,500 ms and the inter-stimulus interval was 500 ms of a fixation cross. The stimulus presentation time was fixed and did not automatically end when the participant responded. Therefore, the length of each block was 60 s. The inter-block interval was 30 s with a blank screen for 15 s, instructions for the next block for 10 s, and a fixation cross for 5 s. The participants were required to press a keyboard key with their right index finger when the stimulus was a target as quickly and accurately as possible. Accuracy (ACC) and reaction time for correct answers (RT) were assessed as performance indices. RT was averaged across RTs of target trials, but RTs of 2 SD or more above or below the mean were excluded because of the susceptibility of outliers due to the low number of target trials. ACC was determined by the average of the percentage of correct responses for target trials (number of correct responses/total number of targets) and the percentage of correct responses for non-target trials (number of correct rejections/total number of non-targets) to equalize the weights of the two trial conditions (Nilsson et al., 2015). In the verbal n-back task, a single Japanese character (hiragana) was presented in a pseudorandom order. In the 0-back condition, the target character was “あ.” In the 1- and 2-back conditions, the target was any character which was same as the character presented one or two trials before, respectively. In the spatial n-back task, eight squares were arranged in a circular pattern. One of the squares was red, and the others were black. In the 0-back condition, the target was the square at the top of the circle when it appeared red. In the 1- and 2-back conditions, the target was any red square that appeared at the same location as the red square presented one and two trials before, respectively. Prior to each task, participants conducted the practice version of the task containing 1 set per condition. All participants were unfamiliar with the task.

### 2.5 fNIRS

#### 2.5.1 fNIRS instruments

As a neuroimaging modality to measure prefrontal activation related to WM, functional near-infrared spectroscopy (fNIRS) has great potential. fNIRS is an optical method that noninvasively monitors the cerebral hemodynamics of oxygenated and deoxygenated hemoglobin species (oxy-Hb and deoxy-Hb, respectively) by measuring changes in the attenuation of near-infrared light passing through tissue (Koizumi et al., 2003;Obrig & Villringer, 2003). fNIRS allows subjects to perform tasks in a natural and comfortable environment without being confined to a small, restricted space. In the fNIRS environment, outside influences on cognitive tasks can be kept to a minimum compared to other neuroimaging methods such as fMRI. Previous studies using fNIRS have revealed increased prefrontal activation with WM load during n-back tasks (Herff et al., 2013;Koike et al., 2013;Vermeij et al., 2014). Additionally, with fNIRS, age-related changes in prefrontal activation have been observed during several cognitive tasks including WM tasks (Heinzel et al., 2012;Hyodo et al., 2012;Tsujii et al., 2010).

Two multichannel fNIRS systems were used in this study: FOIRE-3000 (Shimadzu Corporation, Kyoto, Japan) for older adults and OMM-2000 (Shimadzu Corporation, Kyoto, Japan) for younger adults. Because both apparatuses utilize three wavelengths of near-infrared light (780 nm, 805 nm, and 830 nm), their data are equivalent and can be used for comparisons between the two groups.

#### 2.5.2 fNIRS probe placement

**Fig. 2.**
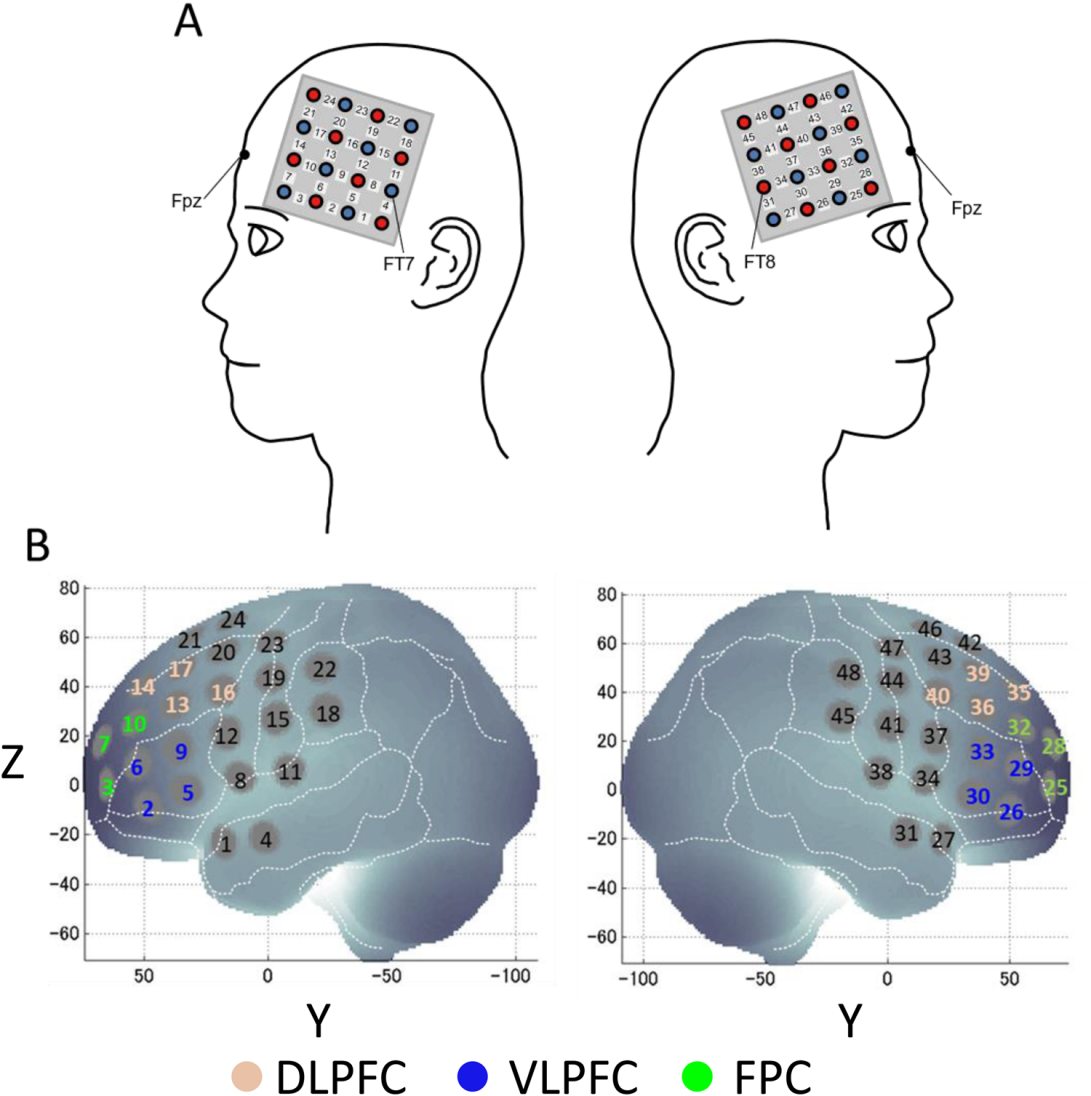
Spatial profiles of fNIRS channels. (A) Side view of probe placement. Channel numbers and FT7 and FT8 of the international 10–10 EEG standard electrode positions are denoted above the corresponding locations. (B) Channel locations estimated by virtual registration depicted in Montreal Neurological Institute (MNI) space. Channel numbers written in beige, blue, and green were located at the left- or right-dorsolateral prefrontal cortex (DLPFC), ventrolateral prefrontal cortex (VLPFC), and frontopolar cortex (FPC), respectively. They were used for data analyses.

The fNIRS probes were set to cover the PFC (Fig. 2A), as in our previous studies (Byun et al., 2014;Hyodo et al., 2012,^2016^;Suwabe et al., 2021). The left probe holder was placed such that probe 5 (between CH4 and CH11) was placed over FT7, with the medial edge of the probe column being parallel to the medial line. The right probe holder was placed symmetrically. Then, we employed virtual registration (Tsuzuki et al., 2007) to register fNIRS data to Montreal Neurological Institute (MNI) standard brain space (Brett et al., 2002;Tsuzuki & Dan, 2014). Briefly, this method allows us to place a virtual probe holder on the scalp by stimulating the holder’s deformation and by registering probes and channels onto reference brains in the MRI database (Okamoto & Dan, 2005;Okamoto et al., 2004). A statistical analysis of the MNI coordinate values for the fNIRS channels was performed to obtain the most likely estimate of the location of given channels for the group of subjects, and the spatial variability associated with the estimation (Singh et al., 2005). Finally, the estimated locations were anatomically labeled using a MATLAB function that reads anatomical labeling information coded in a macro-anatomical brain atlas (Shattuck et al., 2008). Among the 48 channels measured, those channels located outside the PFC (CHs1, 4, 28, and 31) were excluded. Also, channels with poor signal conditions such as extraordinarily abundant high-frequency noise representing insufficient optical signals and abrupt simultaneous changes in oxy-Hb and deoxy-Hb signals suggesting body movements (CHs5, 8, 11, 18, 21, 24, 29, 32, 35, 42, 45, and 48) were removed. Thus, the remaining 22 channels were subjected to further analysis (Fig. 2B).

#### 2.5.3 fNIRS analysis

The optical data were analyzed based on the modified Beer–Lambert Law (Cope et al., 1988) as previously described (Maki et al., 1995). This method allowed us to calculate signals reflecting changes in oxy-Hb, deoxy-Hb, and total hemoglobin (total-Hb) concentrations in units of micro molar-millimeter (μM·mm) (Maki et al., 1995). The sampling rate was set at 10 Hz.

First, channels with a signal fluctuation of 10% or less for oxy- or deoxy-Hb data through the entire duration of the measurement were considered to be defective measurements and were excluded from the analysis. Then, to remove the influence of measurement noise such as breathing, cardiac movement, and so on from the remaining channels, wavelet minimum description length (Wavelet-MDL) was adopted (Jang et al., 2009). Next, the preprocessed oxy-Hb time-series data for each channel in each participant were analyzed using Matlab 2007b (The MathWorks, Inc., Natick, MA, USA) with the tools of the adaptive GLM (Uga et al., 2014), by regressing the data with a linear combination of explanatory variables (i.e., regressors and an error term). Basis functions used for GLM analysis were created by convolving (Equation 2) the boxcar function N ($\tau_{p},\text{t}$) with the hemodynamic response function (HRF) shown inEquation 1(Friston et al., 1998).

$$h\left(\tau_{p},t\right)=\frac{t^{\tau_{p}}e^{-t}}{\left(\tau_{p}\right)!}-\frac{t^{\tau_{p}+\tau_{d}}e^{-t}}{A\left(\tau_{p}+\tau_{d}\right)!}$$ (1)

where t represents a point in the time series,$\tau_{\text{p}}$represents the first peak delay, and$\tau_{\text{d}}$represents the second peak delay. A is the amplitude ratio between the first and second peaks and was set to 6 as in typical fMRI studies.$\tau_{\text{p}}$was adjusted as described below according to the adaptive GLM (Uga et al., 2014), and$\tau_{\text{d}}$was set to 10 s after$\tau_{\text{p}}$as in typical fMRI studies. Basis functions$\text{f(}\tau_{\text{p}},\text{t)}$were generated by convolving the HRF$\text{ h(}\tau_{\text{p}},\text{t)}$with a boxcar functionu(t):

$$\text{f}\left(\tau_{\text{p}},\text{t}\right)=\text{h}\left(\tau_{\text{p}},\text{t}\right)⊗\text{u}\left(\text{t}\right)$$ (2)

where the symbol⊗denotes convolution integral. The basis functions were used to compose each regressor as described below.

The first peak delay$\tau_{p}$was set as a variable changing from 6 to 60 s to yield the optimal HRF. Average β values (task-related amplitudes) across all participants and all analyzed channels were calculated in each n-back condition of younger and older groups, and$\tau_{p}$values with the maximum average β-value in each condition were determined as the respective optimal$\tau_{p}$. The first and second derivatives were included in order to eliminate the influence of noise of individual data. Regressors included were the 60 s task period for each condition (0-/1-/2-back). An example of design matrix X for both verbal and spatial n-back task is shown inSupplementary Figure 1. β values were used as an estimate of the HRF prediction of the oxy- and deoxy-Hb signal. A total of three β values were calculated for each verbal and spatial n-back task (β_0-back_, β_1-back_, β_2-back_). β_0-back_was the β for the task period for the 0-back condition, β_1-back_was for the 1-back condition, and β_2-back_was for the 2-back condition.

### 2.6 Statistical analysis

First, in order to determine controlling variables when comparing RT and brain activity between the older and the younger adults and when evaluating the relationships between aerobic fitness (i.e., VT), task performance (i.e., RT and ACC), and brain activation, we conducted correlation analyses to examine the relationships of VT and task performance with age, sex, and education, which have been identified as potential confounders in previous studies (Erickson et al., 2009;Hyodo et al., 2016;Pliatsikas et al., 2019;Weinstein et al., 2012). Since many correlations had more than small effect sizes (r > 0.1), we used them as covariates for all statistical analyses (Supplementary Table 1).

To clarify any effect of age difference on n-back performance, we performed a two-way mixed ANCOVA using age group (younger/older) as a between-participant factor and memory load (0-/1-/2-back) as a within-participant factor with sex and education as covariates. RT and ACC for each memory load condition were used as dependent variables. Each ANCOVA was followed by post-hoc tests when interaction effect was significant. They included comparisons among three n-back conditions for each age group, and between-age-group comparisons for three n-back conditions resulting in nine contracts for each n-back task. The Holm method was applied for multiple comparison correction. To check whether a speed-accuracy trade-off occurred in this experiment, we conducted a correlation analysis between RT and ACC for each n-back condition.

Next, to clarify prefrontal activation patterns during n-back tasks in younger and older adults, we conducted one-sample t-tests against 0 for oxy- and deoxy-Hb obtained from the adaptive GLM analysis on each channel for each condition of the verbal and spatial n-back tasks. In addition, two-way mixed ANCOVAs were conducted for the oxy- and deoxy-Hb signal values for each CH for each n-back task, with age group (younger/older) as the between-participant factor, memory load (0-/1-/2-back) as the within-participant factor, and sex and years of education as covariates. To control family-wise errors, we used the Holm method (Holm, 1979). In addition, to confirm whether lateralized prefrontal activation patterns were observed during verbal- and spatial-related WM as in previous studies, channels that showed significant activity were averaged for the left and right hemispheres. We conducted paired t-tests between left and right prefrontal activations for each condition in the younger and older adults.

Subsequently, we conducted further analyses to examine the relationships among VT, task performance, and prefrontal activation in older adults. This allowed us to clarify the prefrontal activation patterns specific to older adults with higher fitness and greater n-back-task performance. First, the relationships between VT and performance (ACC and RT) for both types of n-back tasks were examined using Pearson partial correlation analyses controlled by sex, age, and years of education. If RT or ACC for any conditions were related to VT, the relationships among oxy- and deoxy-Hb signal values for the task condition, VT, and task performance were examined using Pearson partial correlation analysis. In addition, when oxy- or deoxy-Hb signal values were related to both VT and task performance, we performed a causal mediation analysis to examine the mediation effect of the activations on the relationship between VT and task performance using the “mediation” package in R. Point estimates with 95% confidence intervals (CI) for the average causal mediation effect (ACME), average direct effect (ADE), and average total effect (ATE) were estimated using a bias-corrected and accelerated bootstrap method with 5,000 resamples. ACME represents the indirect effects of VT on task performance through the oxy- or deoxy-Hb signal values (mediator), and ADE represents the direct effects of VT when the mediator is fixed. The ATE represents the sum of the ACME and the ADE.

Statistical analyses were performed using R 4.0.2 (R Foundation for Statistical Computing, Vienna, Austria) (R Core Team, 2020).

## 3 Results

### 3.1 Behavioral results

**Fig. 3.**
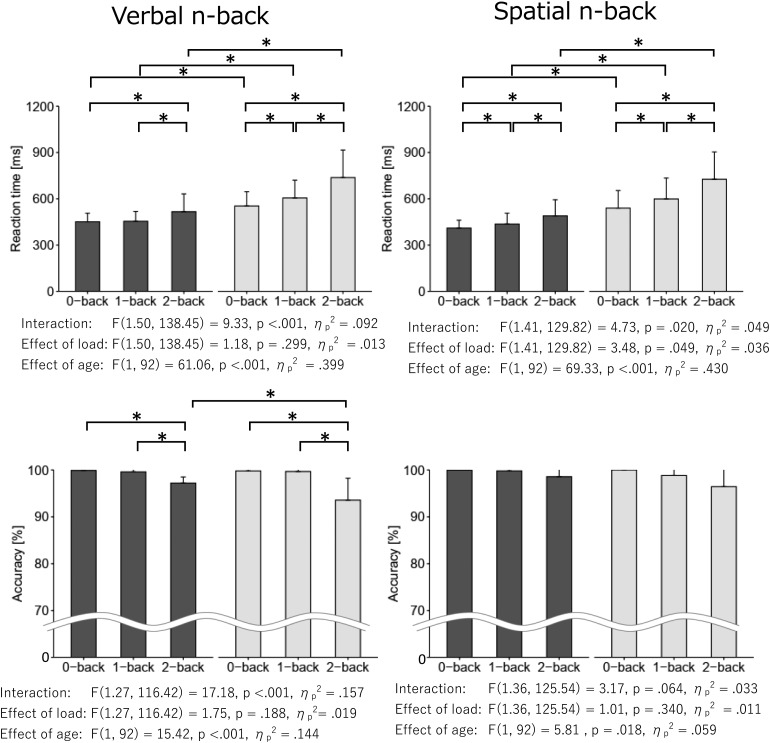
n-back task performance for younger and older adults. Left and right figures show mean and SD of reaction time and accuracy for verbal and spatial n-back tasks, respectively. Results of 2-way ANCOVA between age (younger/older) and memory load (0-, 1-, 2-back) controlled by years of education and sex are shown below each figure. * indicates p < 0.05 in the ANCOVA post-hoc analysis.

Histograms of each n-back task performance for younger and older adults are shown inSupplementary Figure 2.Figure 3shows the n-back task performance for each group and the results of the ANCOVAs, andSupplementary Table 2shows the post-hoc results. The results of the two-way mixed factorial ANCOVAs revealed significant main effects of age group and memory load, and interaction effects between age group and memory load for ACC in both verbal and spatial n-back tasks and between age group and memory load for RT in the verbal n-back task. For ACC in both verbal and spatial n-back tasks and RT in the verbal n-back task, post-hoc analyses were conducted to compare age groups in each task condition and to compare memory load conditions for each age group for each task. In age group comparisons, the RTs of the younger adults were shorter than those of the older adults in every condition. The ACC of the younger adults was higher than that of the older adults in only the verbal 2-back condition. For memory load comparisons, in younger adults, RTs for the verbal 2-back condition were longer than those for the 0- and 1-back conditions. Regarding the spatial n-back task, younger adult RTs for the 2-back condition were longer than those for the 0- and 1-back conditions and those for the 1-back condition were longer than those for the 0-back condition. This was true of the RTs for both the verbal and spatial n-back tasks in older adults. Moreover, ACC for the verbal 2-back condition was lower than that for the 0- and 1-back conditions in both younger and older adults. According to the ACC results for the spatial n-back task, only the main effect of memory load was significant. The post-hoc analysis of main effect of load showed that ACC for the verbal 2-back condition was lower than that for the 0-back condition. Correlation analysis between RT and ACC revealed no speed-accuracy trade-off relationship (Supplementary Fig. 3).

### 3.2 fNIRS results

#### 3.2.1 Search for the adapted HRF

**Fig. 4.**
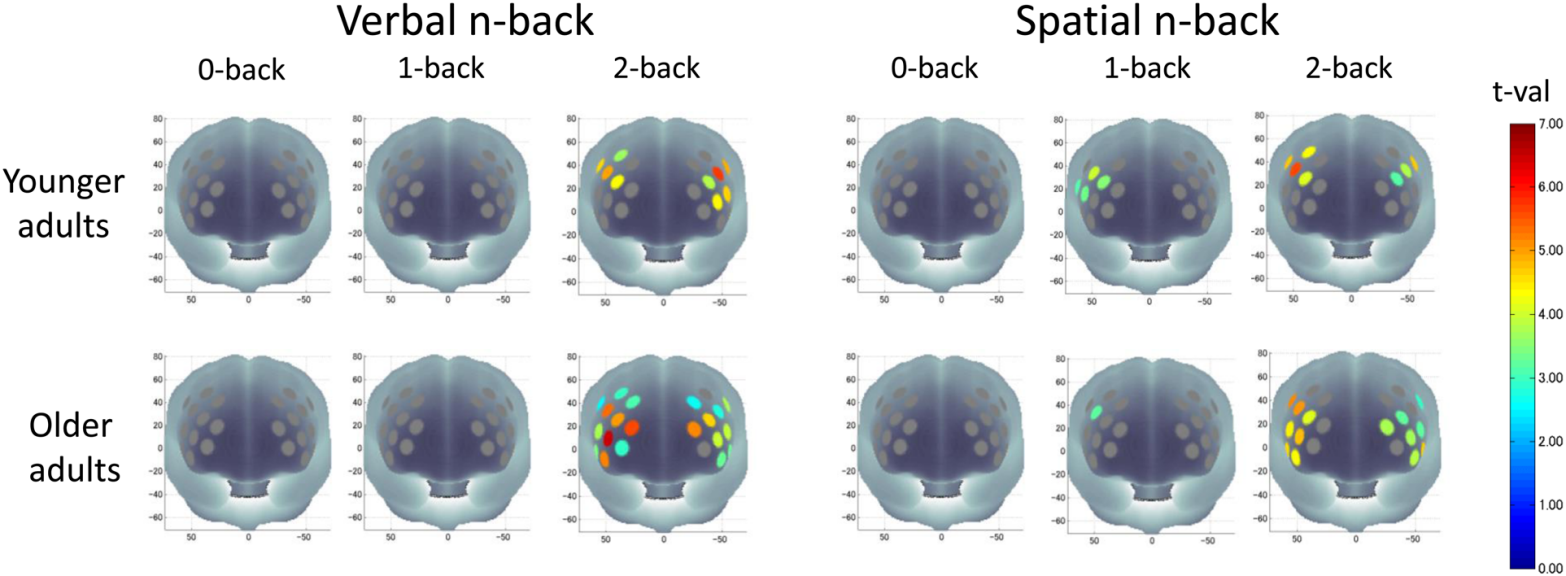
Cortical activation patterns during each n-back task condition (verbal and spatial) in younger (upper row) and older (lower row) adults. Significantly activated channels are colored according to the t-value scale on the right.

The search for the adaptive HRF that best represented the observed changes in oxy- and deoxy-Hb signal during the n-back task is shown inSupplementary Figure 4. For oxy-Hb signal, optimal τ_p_were 6 s, 6 s, and 6 s for younger adults and 6 s, 6 s, and 16 s for older adults in the 0-, 1-, and 2-back conditions of the verbal n-back task. In the 0-, 1-, and 2-back conditions of the spatial n-back task, they were 6 s, 6 s, and 7 s for younger adults and 6 s, 6 s, and 15 s for older adults. For deoxy-Hb signal, optimal τ_p_were 6 s, 7 s, and 28 s for younger adults and 30 s, 8 s, and 18 s for older adults in the 0-, 1-, and 2-back conditions of the verbal n-back task. In the 0-, 1-, and 2-back conditions of the spatial n-back task, they were 6 s, 8 s, and 21 s for younger adults and 6 s, 6 s, and 19 s for older adults.

**Fig. 5.**
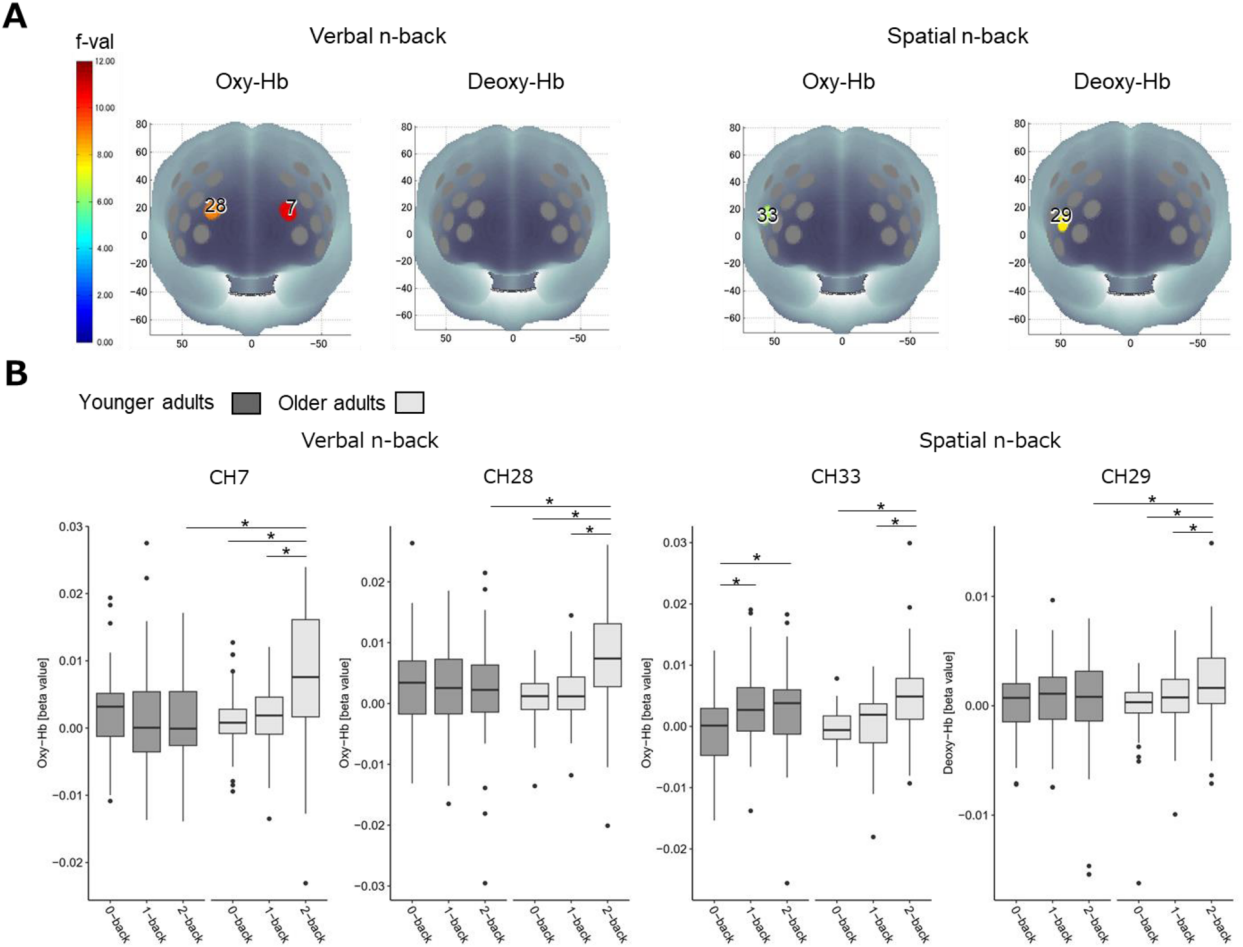
(A) F-maps of oxy-and deoxy-Hb activation reflecting significant interaction effects of group (younger/older) and memory load (0-back/1-back/2-back). (B) Values of oxy-Hb and deoxy-Hb during each condition for each group for channels in which there was interaction (2-way ANCOVA). *Indicates p < 0.05 in the ANCOVA post-hoc analysis.

Therefore, adaptive GLM was performed using these optimal τp. The observed fNIRS timeline data of oxy- and deoxy-Hb signals, which are averaged across all participants and channels, and the optimized HRF for verbal and spatial n-back tasks in younger and older groups are shown inSupplementary Figure 5.

#### 3.2.2 Prefrontal activation pattern during n-back task for each age group

**Fig. 6.**
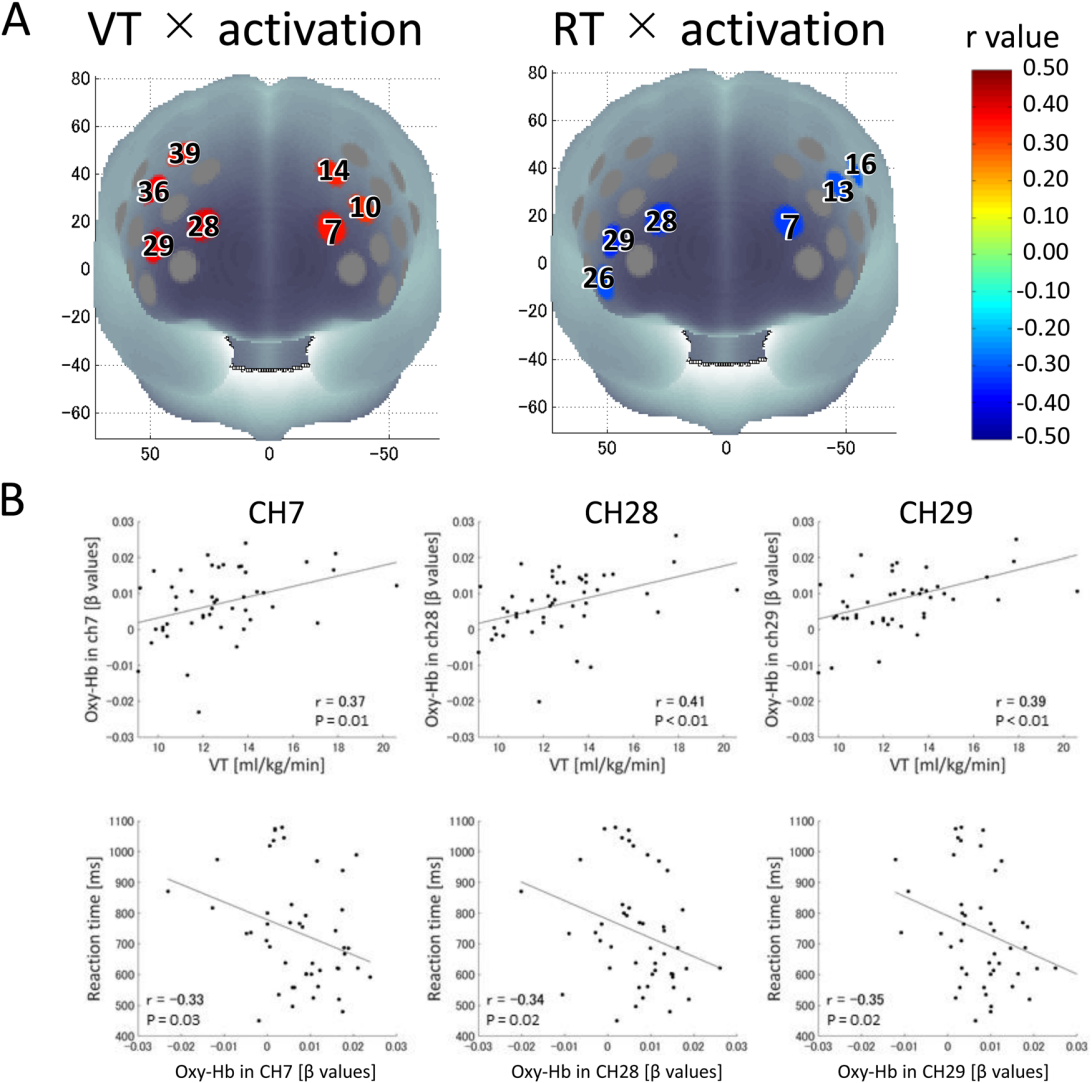
Correlations of oxy-Hb activation during verbal 2-back condition with ventilatory threshold (VT) and reaction time (RT) for verbal 2-back condition. (A) Channels that were significantly associated with VT (left) and RT (right) are colored according to the r-value color scale. CH7, CH28, and CH29 were significantly correlated with both VT and RT. (B) relationships between oxy-Hb in CH7, CH28, CH29, and VT (upper row) and RT (lower row).

Oxy-Hb and deoxy-Hb activation maps for the group analysis with one-sample t-test are shown inFigure 4andSupplementary Figure 6, and the statistical results are shown inSupplementary Tables 3 and 4. Regarding the oxy-Hb activation map, for the verbal n-back task, in younger adults, only the 2-back condition produced significant activation in seven channels on the left PFC and five channels on the right PFC. Similarly, in older adults, only the verbal 2-back conditions produced significant activation in 10 channels on the left PFC and 12 channels on the right PFC. For the spatial n-back task, in younger adults, the 1-back condition produced significant activation in four channels on the right PFC and the 2-back condition produced activation in four channels on the left PFC and five channels on the right PFC. In older adults, the 1-back condition produced significant activation in one channel on the right PFC, and the 2-back condition produced activation in eight channels on the left PFC and eight channels on the right PFC.

Regarding the deoxy-Hb activation map, younger adults showed significant activation in two channels on the left PFC for the verbal 2-back condition and two channels on the right PFC for the spatial 2-back condition. In older adults, for the verbal n-back task, the 0-back condition produced significant activation in one channel on the left PFC and the 2-back condition produced five channels on the right PFC and one channel on the left PFC. For the spatial n-back task, the 1-back condition produced activation in three channels on the left PFC, and the 2-back condition produced activation in five channels on the right PFC and three channels on the left PFC.

These results suggest that older adults recruited more prefrontal regions especially during the verbal and spatial 2-back conditions than did younger adults, particularly in the bilateral VLPFC and FPC.

To confirm the lateralized activation pattern, the channels with significant oxy-Hb activation during both the verbal and spatial 2-back conditions were averaged for left and right hemispheres, respectively. We compared the activation between hemispheres, and found that for the verbal 2-back condition in younger adults, left prefrontal activation was significantly higher (t(48) = 2.06, p = 0.045, d = 0.29) than that on the right side. However, there were no significant differences in brain activation between the left and right hemispheres during the spatial 2-back condition in the younger adults (t(48) = -0.24, p = 0.813, d = -0.03), the verbal 2-back condition in older adults (t(46) = 0.90, p = 0.373, d = 0.13), or the spatial 2-back condition in older adults (t(46) = 1.11, p = 0.274, d = 0.16) (Supplementary Fig. 7).

#### 3.2.3 Age comparison of prefrontal activation

To directly compare the prefrontal activation patterns between age groups, we conducted two-way mixed ANCOVAs for the oxy- and deoxy-Hb signal values of each CH for the verbal and spatial n-back tasks, controlling for sex and years of education. The statistical results of ANCOVA are shown inSupplementary Table 5. The results of the ANCOVAs revealed significant interaction for the oxy-Hb signal values of CHs7 and 28 during the verbal n-back task, and for the oxy-Hb signal values of CH33 and deoxy-Hb signal values of CH29 during the spatial n-back task after Holm correction (Fig. 5). There were no significant main effects of group or memory load. Results of post-hoc analyses are shown inFigure 5andSupplementary Table 6. For age group comparison, oxy-Hb signal values for CHs7 and 28 during the verbal 2-back condition and deoxy-Hb signal values for CH29 for the spatial 2-back condition were significantly higher in the older adults than in the younger adults. For memory load comparison, in the older adults, both oxy- and deoxy-Hb signal values for the 2-back condition were higher than those for the 1-back and 0-back conditions in all channels (CHs7, 28, 29, 33). In younger adults, the oxy-Hb signal values of CH33 during the spatial 1-back and 2-back conditions were higher than that during the 0-back condition.

### 3.3 Relationship between VT, task performance, and prefrontal activation

#### 3.3.1 Correlation analysis

Partial correlation analysis between VT and n-back task performance, controlling for sex, age, and years of education, in older adults revealed a significant negative correlation between VT and RT for the verbal 2-back condition (Table 2,Supplementary Fig. 8).

**Table 2.** Correlation between VT and n-back performance in older adults.

Older adults(n = 47)		RT						AC					
		Verbal			Spatial			Verbal			Spatial		
		0-back	1-back	2-back	0-back	1-back	2-back	0-back	1-back	2-back	0-back	1-back	2-back
VT	r	-0.16	-0.144	-0.32	-0.224	-0.112	-0.007	-0.143	0.177	0.121	-	-0.257	0.051
	p	0.302	0.352	0.034*	0.144	0.471	0.965	0.353	0.251	0.435	-	0.092	0.744

VT: ventilatory threshold, RT: reaction time, ACC: accuracy.

Next, a partial correlation analysis between VT and oxy- and deoxy-Hb signal values during the verbal 2-back condition was performed. Regarding the oxy-Hb signal, there were significant positive correlations of VT with the oxy-Hb signal value of each channel on the left PFC (CHs7, 10, 14, 17) and the right PFC (CHs28, 29, 36, 39) (Fig. 6). Moreover, RT for the verbal 2-back condition had a significant negative correlation with oxy-Hb signal values of channels on the left PFC (CHs7, 13, 16, 17) and the right PFC (CHs26, 28, 29) (Fig. 5). Regarding the deoxy-Hb signal, VT was positively associated with deoxy-Hb signal values of two channels on the left PFC (CHs10, 14) and RT for the verbal 2-back condition was negatively associated with deoxy-Hb signal values of two channels on the left PFC (CHs3, 13). In summary of these correlation analyses, greater oxy-Hb activation in the three channels located on the left FPC (CH7), the right FPC (CH28), and the right VLPFC (CH29) was associated with both higher VT and shorter RT for the verbal 2-back condition. Statistical results of the partial correlation analyses are shown inSupplementary Table 7.

#### 3.3.2 Mediation analyses

**Fig. 7.**
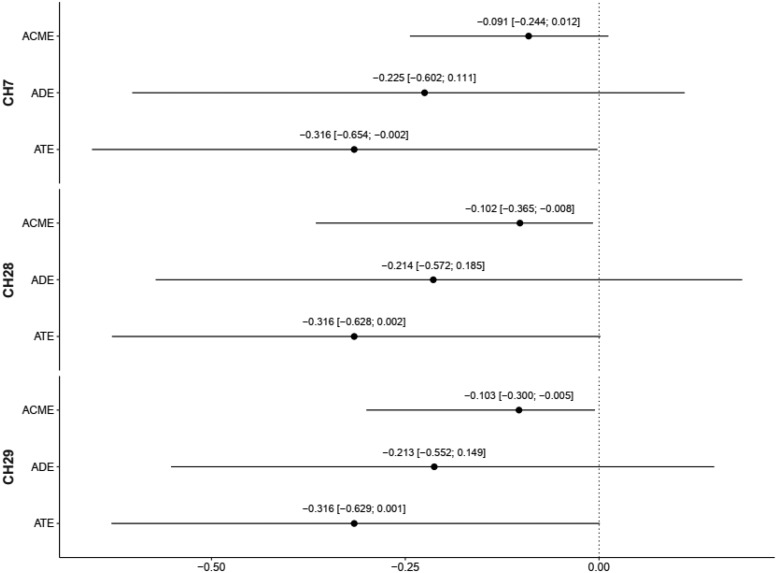
Results of the mediation analysis. Dot and solid line are point estimates and 95% confidence interval (CI) of the standardized effect. Values of the point estimates and 95% CI are shown above each line. ACME: Average Causal Mediation Effect, ADE: Average Direct Effect, ATE: Average Total Effect.

With respect to the oxy-Hb signal values for the verbal 2-back condition at channels 7, 28, and 29, which correlated with both task performance (2-back RT) and VT, mediation analyses were performed adjusting for age, sex, and years of education.Figure 7shows the results of the mediation analyses. The 95% CI of the ACME for the oxy-Hb signal values of CH28 (-0.365 to -0.008) and CH29 (-0.300 to -0.005) did not include zero. These results suggest that oxy-Hb activation of the right FPA (CH28) and right VLPFC (CH29) may mediate the relationship between VT and RT for verbal 2-back RT.

## 4 Discussion

In this study, we aimed to clarify the prefrontal activation patterns that are associated with the relationship between aerobic fitness and WM in older adults, focusing on age-related overactivation. We confirmed the age-related decline in n-back task performance and recruitment of more prefrontal areas during verbal and spatial 2-back conditions by comparison to these factors in younger adults. Furthermore, the additional activation in the right FPC and VLPFC exhibited a mediation effect on the relationship between VT and RT for the verbal 2-back condition in older adults. These findings suggest that compensatory prefrontal activation could be one of the neural mechanisms underpinning greater WM performance in older adults with higher aerobic fitness levels.

### 4.1 Age-based differences in task performance and prefrontal activation

In the present study, we confirmed that, compared to younger adults, older adults perform worse on n-back tasks, as seen by delayed RT and reduced ACC; this is especially so for the 2-back conditions, which require a higher memory load which, in turn, entails the recruitment of more prefrontal regions, particularly among the channels located in the bilateral VLPFC and FPC. The decline in n-back performance with age observed in this study is consistent with a recent meta-analysis that revealed large and significant age differences observed when the n in an n-back task is greater than 2 (Bopp & Verhaeghen, 2018).

Consistent with previous studies showing that younger adults exhibit predominantly left hemispheric activation during verbal WM tasks and bilateral activation during spatial WM tasks (Cona & Scarpazza, 2019;Owen et al., 2005;Thomason et al., 2009), we found left prefrontal activation during the verbal 2-back condition and bilateral activation during the spatial 2-back condition in younger adults. Furthermore, in older adults, the predominant hemispheric activation during the verbal 2-back condition disappeared. This is consistent with the HAROLD model (Cabeza, 2002). Moreover, compared to younger adults, older adults showed additional or greater activation mainly on the VLPFC and FPC in the higher WM load 2-back conditions. While this age-related overactivation was mainly analyzed focusing on oxy-Hb signal, which produces higher signal strength (Strangman et al., 2002), the overall tendency was also confirmed with deoxy-Hb signal. Previous studies have shown age-related recruitment in extended PFC regions during cognitive tasks similar to our 2-back conditions (Cabeza, 2002;Reuter-Lorenz et al., 2000;Saliasi et al., 2014).Saliasi et al. (2014)found that, compared to younger adults, older adults had greater neural activation in the bilateral VLPFC for n-back conditions in which n was more than two. On the other hand, unlike the results of the present study, it has been reported that prefrontal activation during high WM load conditions is reduced in older adults compared to younger adults (Mattay et al., 2006). However, in this case, the Compensation-Related Utilization of Neural Circuits Hypothesis (CRUNCH) should be considered (Reuter-Lorenz & Cappell, 2008). CRUNCH indicates that brain activity increases with the cognitive load of the task at all ages. Older adults recruit more neural resource than younger adults under low or intermediate loads to compensate for neural inefficiency, but when the WM load exceeds WM capacity, the compensatory mechanism cannot work effectively and results in less activation. From this perspective, the lack of greater prefrontal activation for the 1-back condition in older adults compared to younger adults may indicate that the WM load of the 1-back condition was not large enough to produce increased prefrontal activation for the older adults in this study. Moreover, it is possible that the age-related extended and greater recruitment in the PFC for the 2-back conditions in this study was due to the fact that the 2-back condition was still easy for the younger participants and still within the WM capacity of the older participants.

### 4.2 Association between aerobic fitness and task performance in older adults

To explore the brain activation associated with higher task performance in older adults with higher aerobic capacity, we first examined the behavioral task performance associated with aerobic capacity. The correlation analysis showed that VT was negatively correlated with RT for the verbal 2-back task in older adults. This is consistent with the results of previous studies reporting that aerobic fitness is related to cognitive function under high-load, difficult conditions (Prakash et al., 2011;Weinstein et al., 2012). Unlike RT, however, there were non-significant relationships between VT and ACC for the n-back tasks in this study. One reason for this result may be that more than 85% of older participants had an ACC higher than 90%, and there were ceiling effects in ACC across the participants in this study. Another reason may be that aerobic fitness is related to WM processes related to RT (the speed at which information can be retrieved from WM) (Jensen, 1993). Moreover, RTs for n-back tasks have been reported to be related to IQ (Gevins & Smith, 2000;Hockey & Geffen, 2004), which may reflect that global intelligence is related to aerobic fitness.

Unlike with the verbal n-back performance, there was no significant relationship between VT and spatial n-back performance. This is inconsistent with previous studies reporting a positive relationship between V̇o_2peak_and RT or ACC during spatial WM tasks (Erickson et al., 2009;Oberlin et al., 2016;Szabo et al., 2011;Weinstein et al., 2012). The difference in the type of spatial WM tasks could account for apparent differences between previous studies and ours. The spatial WM tasks used in previous studies were short-term memory tasks that did not require participants to manipulate and update stored information. These short-term spatial memory tasks have been reported to be associated with hippocampal volume (Erickson et al., 2009;Szabo et al., 2011). Since exercise has a positive effect on the structure and functional connectivity of the hippocampus in older adults (Wilckens et al., 2021;Won et al., 2021), hippocampus-dependent spatial WM tasks might be more closely associated with aerobic fitness. Recent studies have reported that aerobic fitness is not associated with object n-back task performance (Predovan et al., 2021) or with composite score of forward digit span, backward digit span, and letter-number sequencing tasks (España-Irla et al., 2021) in older adults. Further research using a variety of working memory tasks is needed to explore the comprehensive relationship between aerobic fitness and WM.

### 4.3 Association of prefrontal activation with aerobic fitness and task performance in older adults

Since we found a relationship between VT and RT for the verbal 2-back condition, we will discuss the relationship between prefrontal activation for the verbal 2-back condition with regard to RT and VT in the older adults. The verbal 2-back condition evoked brain activation on channels located in the bilateral FPC and right VLPFC, and these were correlated with VT and RT in older adults. Moreover, mediation analysis indicated that these oxy-Hb activations may have a mediating effect on the relationship between VT and RT (95% confidence interval does not include zero). These channels did not show significant activation in younger adults and were more strongly activated in older adults. These results suggest that older adults with higher aerobic fitness might recruit more compensatory prefrontal areas than those with lower aerobic fitness, which, in turn, contributes to improved WM performance. The positive relationship between age-related extended prefrontal activation and working-memory performance found in this study is consistent with previous neuroimaging studies.Sala-Llonch et al. (2012)found that older adults who had higher n-back accuracy scores for a verbal 3-back condition also had greater activation in the right inferior frontal gyrus (corresponding to the VLPFC) than did older adults with lower performance.Saliasi et al. (2014)have also reported that accuracy for a verbal n-back task was associated with activation within the VLPFC and that older adults had more activation than did younger adults. Our results are also consistent with those ofAgbangla et al. (2019), who reported that higher-fitness older adults had greater bilateral activation compared to lower-fitness older adults during a verbal 3-back condition. In addition to the finding ofAgbangla et al. (2019), the present study further examined neural mechanisms by analyzing multi-channel fNIRS data, and found that the VLPFC and FPC could be involved in the relationship between aerobic fitness and working memory performance in older adults. The VLPFC and FPC play crucial roles in the WM process. It has been confirmed that the VLPFC maintains verbal information during WM tasks (Owen et al., 2005;Reuter-Lorenz et al., 2000) and that the FPA is related to complicated, multitasking processes (Koechlin et al., 1999). Therefore, these findings support the hypothesis that older adults with a higher fitness level performed the WM task with shorter RTs by more efficiently maintaining and applying multitask neural processes during the n-back tasks.

The underlying mechanism of the relationship between aerobic fitness and brain activation could be considered in terms of brain structure and molecular pathways. Animal studies have revealed that regular exercise enhances neural and synaptic plasticity, including neurogenesis, and angiogenesis, through the production of nerve-growth factors such as brain-derived neurotrophic factor and insulin-like growth factor-1 (Ding et al., 2006;Inoue et al., 2015;Okamoto et al., 2012;van Praag et al., 1999). Human studies have also found a positive relationship between aerobic fitness and brain structure, including volume of gray and white matter (Colcombe et al., 2006;Weinstein et al., 2012) and white matter integrity (Johnson et al., 2012;Oberlin et al., 2016) in older adults. Older adults with greater neural capacity could recruit sufficient neural resources to meet cognitive demands (Cabeza et al., 2018). Thus, older adults with higher aerobic capacity could, due to a larger neural capacity, have a greater ability to recruit more prefrontal regions in order to perform WM tasks with a higher cognitive demand.

## 5 Limitations

There are some limitations in this study. First, because of the cross-sectional design, causal relationships among aerobic fitness, brain activation, and WM performance are still unknown. Therefore, for further study, whether or not aerobic fitness that has been increased via an exercise intervention leads to greater compensatory brain activation and cognitive performance should be examined. On this point, we should also consider the possibility that the exercise intervention may improve neural efficiency of the primary network, and, as a result, reduce the need for compensatory prefrontal activation (Byun et al., 2023). Second, we measured prefrontal activation in younger and older adults using different fNIRS apparatuses. Although these apparatuses utilize the same principles to calculate oxy- and deoxy-Hb data and are comparable, minute differences could affect the detection, and thus comparison, of prefrontal activation between the groups. Third, these fNIRS apparatuses did not have short-separation channels. Although some systemic effects (such as respiration and heartbeat) were reduced through pre-processing tools such as Wavelet-MDL, skin blood flow could affect the fNIRS data. However, since we observed lateralized activation patterns during the verbal 2-back task in younger adults (Supplementary Fig. 7), where we would expect systemic effects to exhibit uniform activations among channels (Noah et al., 2021), we believe that task-related cortical activation was, in fact, detected. Fourth, a sampling bias may have occurred because the recruitment strategies were different for older and younger participants, limiting the comparability of the groups. Fifth, the sample size for this study was limited, and, in particular, possibly not large enough to conduct a conclusive mediation analysis with the effect size of this study in general (Fritz & Mackinnon, 2007). Further research with a larger sample is needed to increase the robustness and generalizability of the results. Sixth, although the cognitive task protocol was designed specifically to account for fatigue, the limited number of trials for the n-back tasks (10 target trials for each condition) may cause problems with the reliability of the task performance scores. Seventh, n-back trials were presented in ascending order (0-back → 1-back → 2-back) rather than in random order to avoid confusion and reduce switch costs, particularly in older adults (Kray & Lindenberger, 2000). Therefore, it is possible that fatigue and habituation effects may have occurred as the session progressed and WM load increased. This may have affected task performance and prefrontal activation. Last, it might be possible that brain regions other than the PFC are associated with aerobic fitness and WM performance in older adults. Previous studies have reported that the connectivity between prefrontal and parietal cortices is important for WM performance (Nagel et al., 2011), and that age-related increase in this connectivity has been observed during n-back tasks (Sala-Llonch et al., 2012). Interestingly, a recent study in younger adults showed aerobic fitness was associated with the fronto-parietal network (Ishihara et al., 2020). For a deeper understanding of the neural mechanisms involved, further study should focus on the functional connectivity between the PFC and the parietal cortex.

## 6 Conclusion

This study confirmed the age-related decline in WM task performance and bilateral extended prefrontal recruitment during high WM load conditions of verbal and spatial n-back tasks as with previous studies. In addition, we found that aerobic fitness was associated with RT for verbal 2-back condition in older adults, and this relationship was mediated by age-related overactivation in the right FPA and VLPFC. These results suggest that the association between aerobic fitness and WM ability differs depending on the type of WM task, and that older adults with higher aerobic fitness have greater verbal WM performance by having the ability to recruit more compensatory activation in prefrontal regions. This study provides insight into the neural mechanisms of the relationship between aerobic fitness and WM in light of functional brain activation.

## Supplementary Material

Supplementary Material

## Data Availability

The group-level data and code that were used in this study are available to researchers from the corresponding author upon reasonable request, given appropriate ethical and data-sharing agreements. The individual data in this article are not readily available because we do not have permission from the participants to share the raw data. Requests to access datasets should be directed tok-hyodo@my-zaidan.or.jp.
