# Supplementary material for: Neural mechanisms of the relationship between aerobic fitness and working memory in older adults: An fNIRS study
Source: Imaging Neurosci (Camb). 2024 May 10;2:imag-2-00167. doi: 10.1162/imag_a_00167 (PMC12247563; doi:10.1162/imag_a_00167)
Supplement: Supplementary Material [file imag_a_00167-supp.pdf]

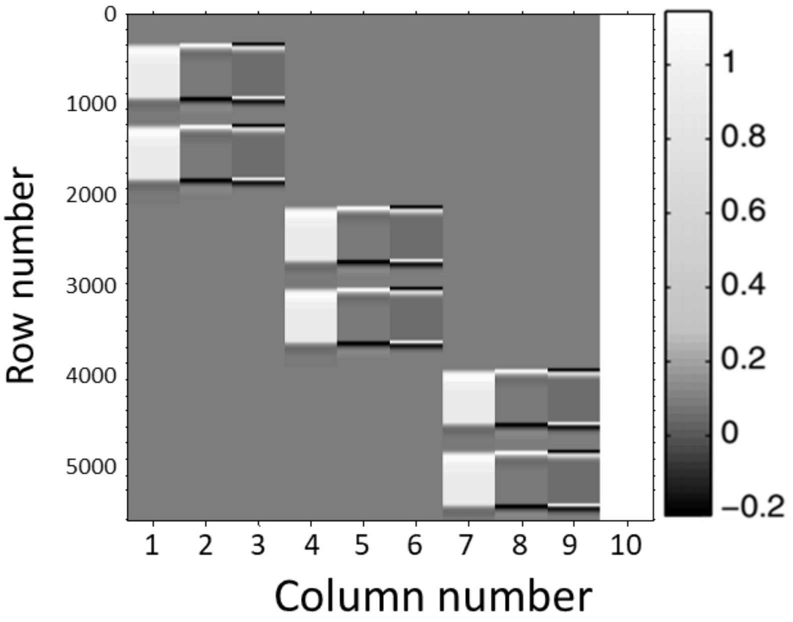

Supplementary Figure 1. Example of design matrix X. As described in Section 2.5.3, a peak delay was set as τ_p = 6 s. The row number indicates the number of time points. Columns 1, 2, and 3 respectively represent the HRF of the task period for the 0-back condition and the first and second derivatives. Columns 4, 5, and 6 respectively represent the HRF of the task period for the 1-back condition and the first and second derivatives. Columns 7, 8, and 9 respectively represent the HRF of the task period for 2-back condition and the first and second derivatives. Column 10 represents the constant.

Supplementary Table 1. Relationships of possible covariates with VT and n-back task performance in older adults

n = 47		Verbal						Spatial					
		ACC			RT			ACC			RT		
Covariates	VT	0-back	1-back	2-back	0-back	1-back	2-back	0-back	1-back	2-back	0-back	1-back	2-back
Age	0.12	0.19	0.01	-0.42	0.00	0.18	0.26	-	-0.17	-0.39	0.02	0.22	0.41
Sex	0.22	0.08	0.03	-0.11	0.13	0.11	0.05	-	0.17	0.06	0.00	-0.10	0.12
Education	-0.07	0.02	0.22	0.02	0.07	0.16	0.10	-	0.26	-0.02	0.15	0.00	-0.05

Values are correlation coefficients obtained from Pearson correlation analysis.

VT: ventilatory threshold, RT: reaction time, ACC: accuracy

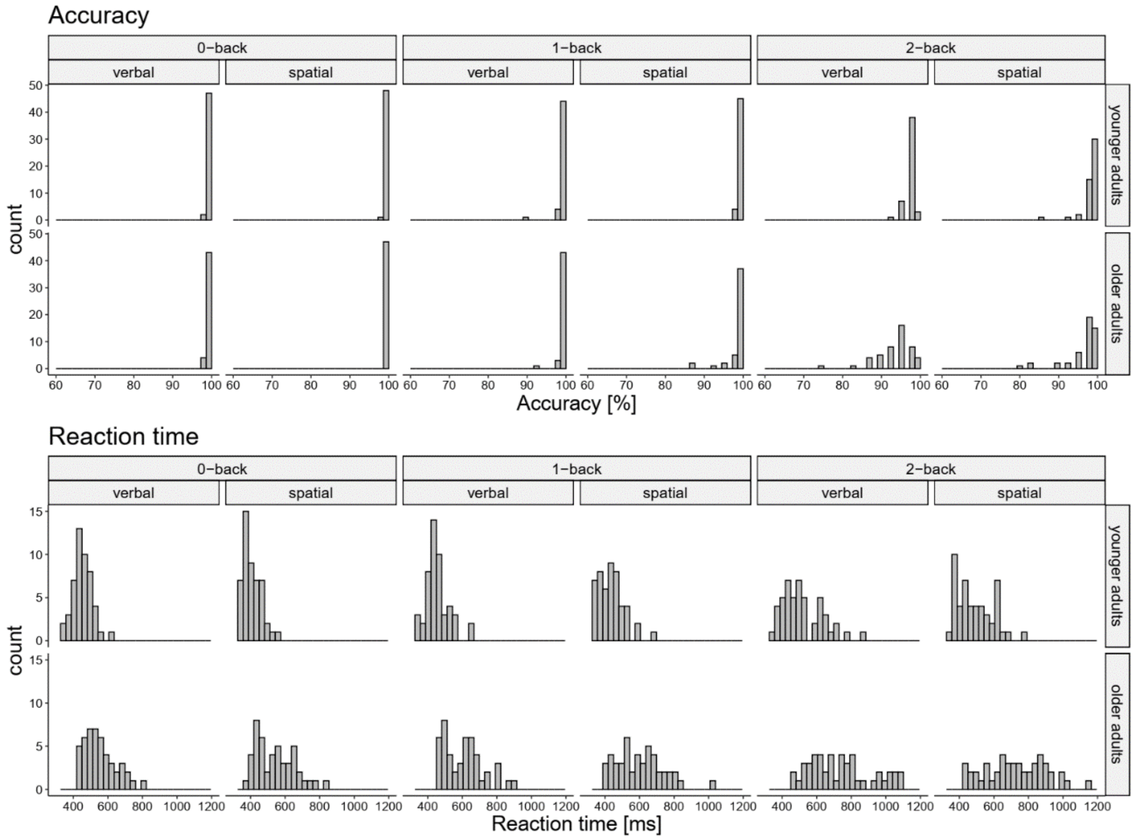

Supplementary Figure 2. Histograms of n-back task performance for younger and older adults

Supplementary Table 2. Post-hoc comparisons after ANCOVA for n-back task performance

Simple main effect of memory load (0-back/1-back/2-back)

			0-back – 1-back			0-back – 2-back			1-back – 2-back		
Group	Task	Variable	t	P_{holm}	d	t	P_{holm}	d	t	P_{holm}	d
Younger adults	Verbal	ACC	1.669	0.394	0.238	**5.181**	< 0.001	0.740	**4.014**	0.001	0.573
		RT	-0.097	0.923	-0.014	**-3.166**	0.004	-0.452	**-3.395**	0.003	-0.485
	Spatial	ACC	1.160	0.748	0.166	2.497	0.086	0.357	1.633	0.424	0.233
		RT	**-2.652**	0.009	-0.379	**-3.869**	0.001	-0.553	**-2.698**	0.017	-0.385
Older adults	Verbal	ACC	0.088	0.930	0.013	**11.386**	< 0.001	1.661	**10.290**	< 0.001	1.501
		RT	**-4.412**	< 0.001	-0.644	**-8.470**	< 0.001	-1.235	**-6.531**	< 0.001	-0.953
	Spatial	ACC	**2.824**	0.041	0.412	**5.777**	< 0.001	0.843	**3.709**	0.003	0.541
		RT	**-4.288**	< 0.001	-0.625	**-7.417**	< 0.001	-1.082	**-5.604**	< 0.001	-0.817

Simple main effect of group (younger adults/older adults)

		Younger adults - Older adults								
		0-back			1-back			2-back		
Task	variable	t	P_{holm}	d	t	P_{holm}	d	t	P_{holm}	d
Verbal	ACC	0.479	1.000	0.098	-0.954	1.000	-0.195	**4.449**	< 0.001	0.908
	RT	**-5.950**	< 0.001	-1.215	**-7.290**	< 0.001	-1.488	**-6.330**	< 0.001	-1.292
Spatial	ACC	-0.891	0.375	-0.182	1.050	0.593	0.214	2.222	0.144	0.454
	RT	**-6.729**	< 0.001	-1.374	**-6.347**	< 0.001	-1.296	**-6.640**	< 0.001	-1.356

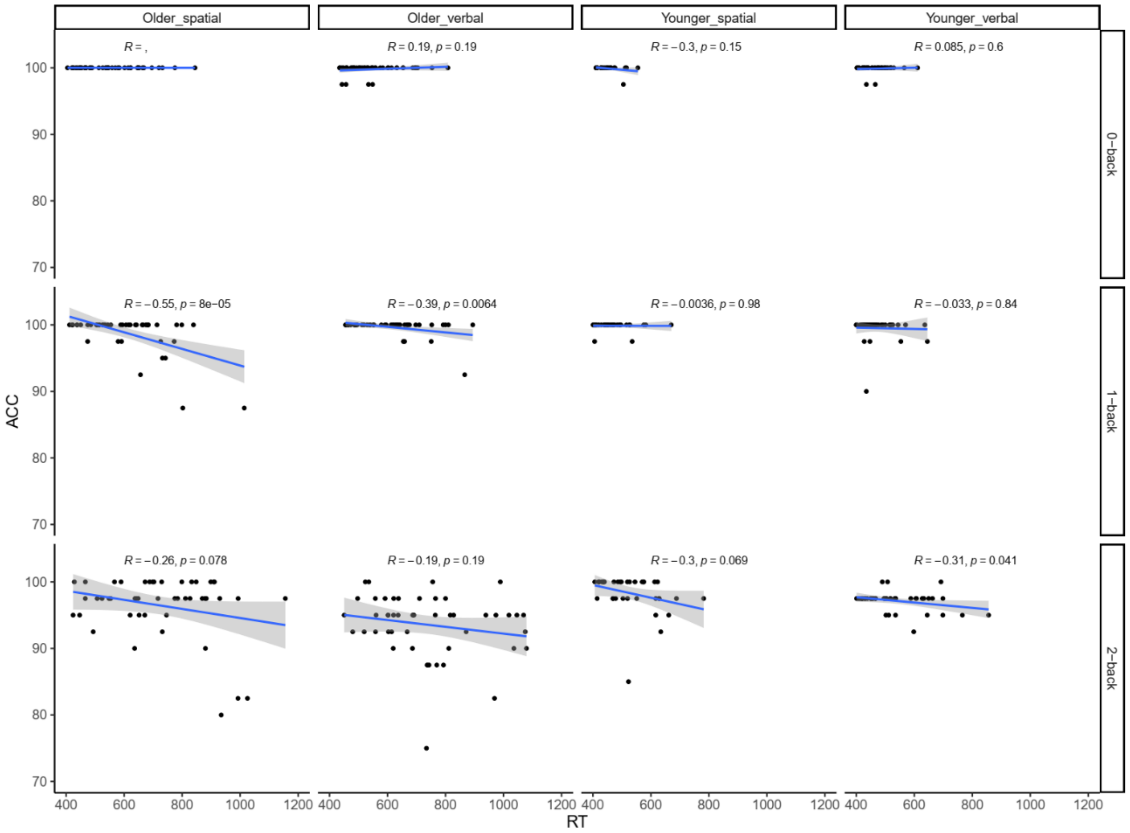

Supplementary Figure 3. Relationship between reaction time and accuracy for each n-back task condition in younger and older adults. Pearson correlation coefficient and p-values are shown in each figure.

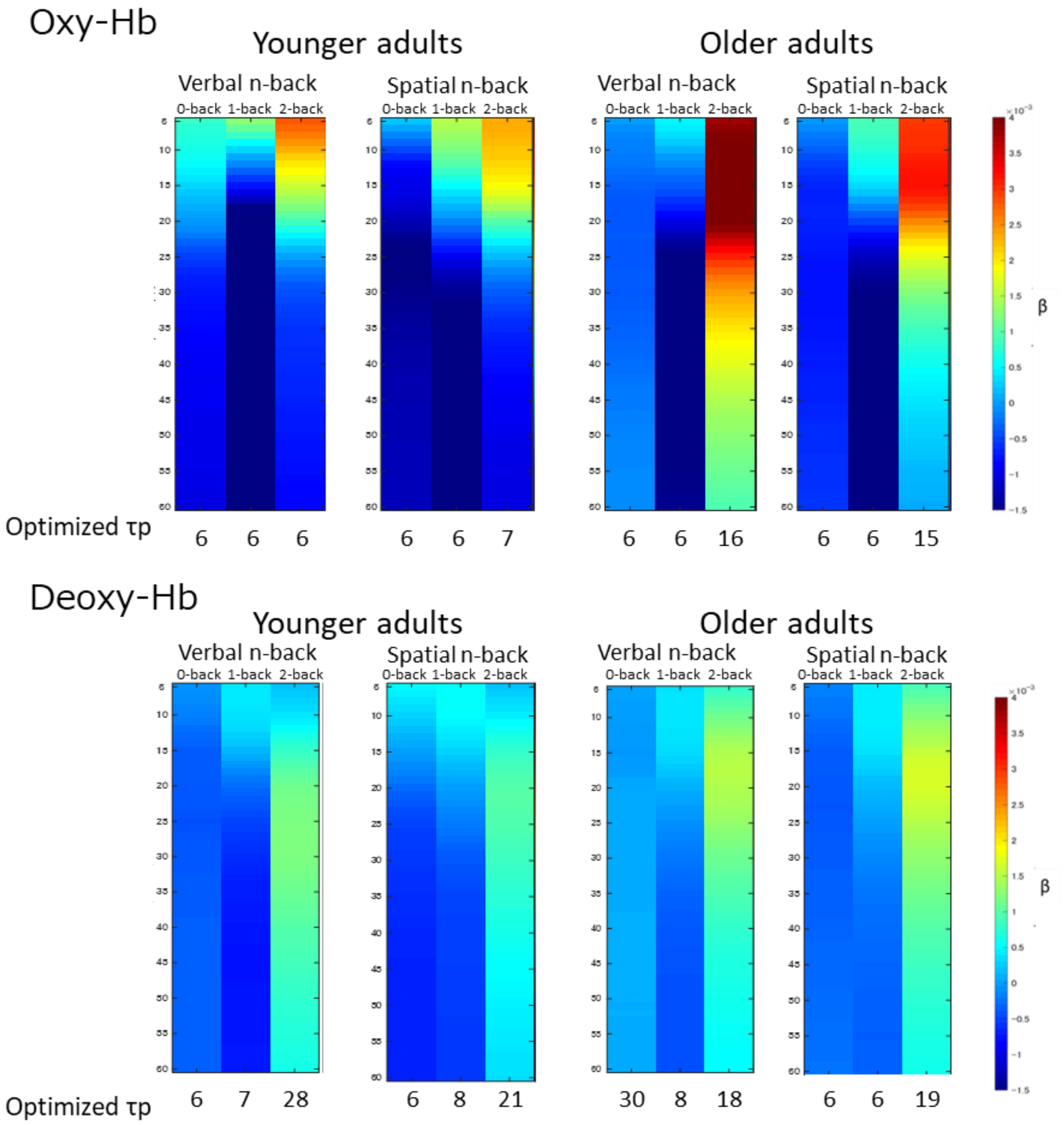

Supplementary Figure 4. Average β-values across all participants and channels calculated for various τ_p values for each condition of verbal and spatial n-back tasks in younger and older adults. Magnitude of the β-values are indicated by the color scale. Optimal τ_p values, which gave the maximum β-values, are presented under each column.

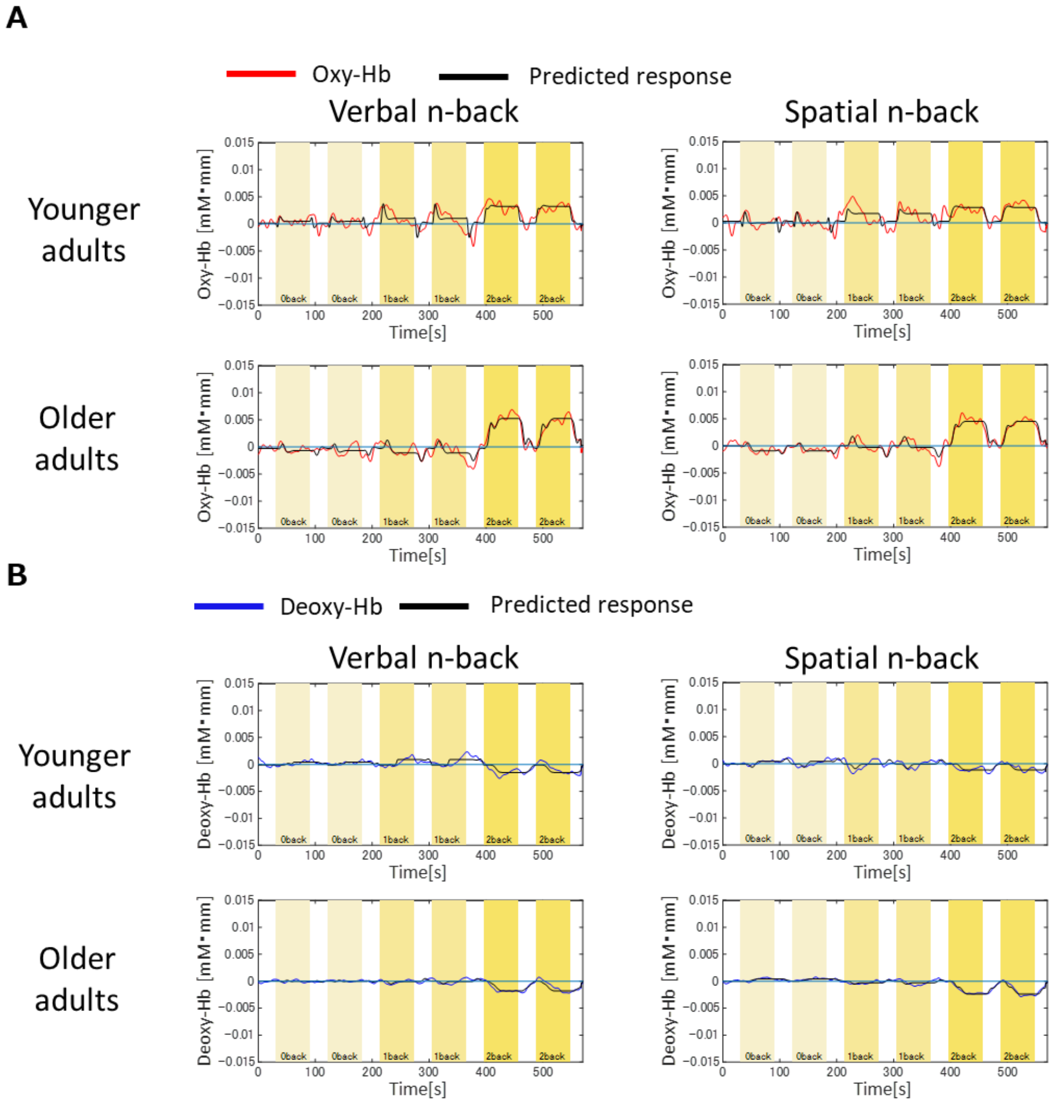

Supplementary Figure 5. The observed timeline data for (A) oxy-Hb and (B) deoxy-Hb signals and predicted responses with optimized regressors for two n-back tasks in both groups. The red and blue lines indicate the observed timelines for oxy- Hb signal and the black lines indicate the predicted time responses with optimized regressors, which were calculated using the optimal τ_p values for 2-back condition. The observed timeline data are the grand-average data across all participants and ROI channels.

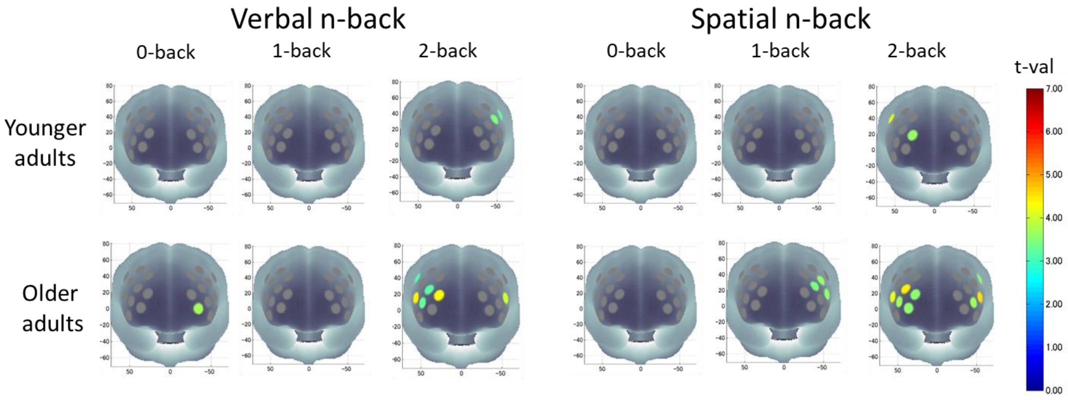

Supplementary Figure 6. Deoxy-Hb activation patterns for each condition of verbal and spatial n-back tasks in younger (upper row) and older (lower row) adults. Significantly activated channels are colored according to the t-value scale on the right.

Supplementary Table 3. One-sample t-test for oxy-Hb

Verbal n-back task in younger adults (n = 49)

	0-back			1-back			2-back		
CH	t	P_{holm}	d	t	P_{holm}	d	t	P_{holm}	d
2	0.407	1	0.058	-0.636	1	-0.091	0.373	1	0.053
3	0.694	1	0.099	0.493	0.624	0.07	0.788	1	0.113
5	-0.895	1	-0.128	-1.199	1	-0.171	0.921	1	0.132
6	2.192	0.566	0.313	2.083	0.724	0.298	**4.41**	0.001	0.63
7	2.71	0.205	0.387	0.982	1	0.14	1.142	1	0.163
9	0.558	1	0.08	1.475	1	0.211	**4.672**	<0.001	0.667
10	2.263	0.508	0.323	3.132	0.065	0.447	**3.862**	0.005	0.552
13	0.569	1	0.081	1.978	0.752	0.283	**5.751**	0	0.822
14	2.346	0.462	0.335	2.916	0.113	0.417	1.577	0.968	0.225
16	-0.154	1	-0.022	2.142	0.671	0.306	**4.942**	0	0.706
17	1.24	1	0.177	1.739	1	0.248	2.524	0.195	0.361
25	-0.411	1	-0.059	-0.793	1	-0.113	0.343	1	0.049
26	-0.338	1	-0.048	-1.439	1	-0.206	-0.261	0.795	-0.037
28	2.384	0.443	0.341	2.567	0.268	0.367	1.82	0.75	0.26
29	0.247	1	0.035	1.507	1	0.215	2.335	0.262	0.334
30	-1.102	1	-0.157	-0.978	1	-0.14	0.726	1	0.104
32	2.317	0.471	0.331	2.494	0.306	0.356	**4.355**	0.001	0.622
33	-0.397	1	-0.057	1.46	1	0.209	1.678	0.898	0.24
35	1.59	1	0.227	1.581	1	0.226	2.405	0.241	0.344
36	0.448	1	0.064	1.98	0.801	0.283	**5.004**	0	0.715
39	0.121	1	0.017	2.001	0.818	0.286	**3.71**	0.008	0.53
40	-0.042	0.967	-0.006	1.862	0.893	0.266	**4.63**	0	0.661

Spatial n-back task in younger adults (n = 49)

	0-back			1-back			2-back		
CH	t	P_{holm}	d	t	P_{holm}	d	t	P_{holm}	d
2	-1.605	1	-0.229	-0.331	1	-0.047	0.294	1	0.042
3	-0.872	1	-0.125	-0.166	1	-0.024	-0.114	0.909	-0.016
5	-1.291	1	-0.184	0.084	0.933	0.012	0.828	1	0.118
6	0.158	1	0.023	1.399	1	0.2	1.972	0.489	0.282
7	0.459	1	0.066	0.465	1	0.066	0.913	1	0.13
9	0.04	0.968	0.006	0.523	1	0.075	2.153	0.399	0.308
10	1.499	1	0.214	1.956	0.788	0.279	**3.23**	0.036	0.461
13	1.375	1	0.196	2.275	0.411	0.325	**3.835**	0.006	0.548
14	2.015	1	0.288	1.829	0.883	0.261	1.389	1	0.198
16	0.106	1	0.015	1.218	1	0.174	**4.723**	<0.001	0.675
17	0.97	1	0.139	1.894	0.835	0.271	2.559	0.164	0.366
25	0.288	1	0.041	1.055	1	0.151	1.537	1	0.22
26	0.505	1	0.072	0.654	1	0.093	0.896	1	0.128
28	0.358	1	0.051	2.347	0.37	0.335	2.046	0.462	0.292
29	0.934	1	0.133	2.82	0.118	0.403	3.011	0.062	0.43
30	-1.105	1	-0.158	0.871	1	0.124	1.507	0.966	0.215
32	1.329	1	0.19	**3.564**	0.018	0.509	**4.146**	0.002	0.592
33	-0.898	1	-0.128	**3.4**	0.027	0.486	2.658	0.138	0.38
35	0.962	1	0.137	3.118	0.058	0.445	2.879	0.083	0.411
36	0.27	1	0.039	**4.021**	0.004	0.574	**5.635**	<0.001	0.805
39	-0.15	1	-0.021	3.036	0.07	0.434	**4.38**	0.001	0.626
40	-0.144	1	-0.021	1.823	0.82	0.26	**4.743**	<0.001	0.678

Bold indicates statistical significance.

(continued)

Verbal n-back task in older adults (n = 47)

CH	0-back			1-back			2-back		
	t	P_{holm}	d	t	P_{holm}	d	t	P_{holm}	d
2	0.893	1	0.13	0.229	1	0.033	**3.337**	0.015	0.487
3	-0.244	1	-0.036	-0.436	1	-0.064	2.024	0.098	0.295
5	0.093	1	0.014	0.558	1	0.081	**3.378**	0.015	0.493
6	0.48	1	0.07	2.761	0.182	0.403	**4.015**	0.003	0.586
7	1.479	1	0.216	2.176	0.625	0.317	**5.191**	<0.001	0.757
9	-1.004	1	-0.146	-0.287	1	-0.042	**3.955**	0.004	0.577
10	-0.486	1	-0.071	1.494	1	0.218	**4.663**	<0.001	0.68
13	-1.131	1	-0.165	-0.599	1	-0.087	**2.846**	0.033	0.415
14	-1.109	1	-0.162	-0.372	1	-0.054	**2.702**	0.038	0.394
16	-0.676	1	-0.099	-0.493	1	-0.072	**3.796**	0.005	0.554
17	-1.985	1	-0.29	-1.424	1	-0.208	1.53	0.133	0.223
25	-0.017	0.986	-0.003	0.47	1	0.068	**2.988**	0.027	0.436
26	-0.273	1	-0.04	1.351	1	0.197	**5.219**	<0.001	0.761
28	1.47	1	0.214	2.583	0.262	0.377	**5.644**	<0.001	0.823
29	2.278	0.603	0.332	2.511	0.296	0.366	**6.496**	<0.001	0.948
30	-0.645	1	-0.094	1.119	1	0.163	**3.497**	0.012	0.51
32	0.612	1	0.089	2.17	0.598	0.317	**5.052**	0	0.737
33	-0.162	1	-0.024	0.933	1	0.136	**3.803**	0.005	0.555
35	-0.512	1	-0.075	0.279	1	0.041	**3.158**	0.022	0.461
36	1.362	1	0.199	2.603	0.26	0.38	**5.399**	0	0.788
39	-1.655	1	-0.241	0.185	1	0.027	**3.038**	0.027	0.443
40	-0.85	1	-0.124	-0.127	0.9	-0.018	**2.613**	0.036	0.381

Spatial n-back task in older adults (n = 47)

CH	0-back			1-back			2-back		
	t	P_{holm}	d	t	P_{holm}	d	t	P_{holm}	d
2	0.743	1	0.108	1.198	1	0.175	**3.876**	0.005	0.565
3	0.775	1	0.113	1.471	1	0.215	1.161	0.756	0.169
5	-0.77	1	-0.112	-0.114	1	-0.017	**4.711**	<0.001	0.687
6	0.766	1	0.112	1.133	1	0.165	**3.762**	0.006	0.549
7	0.632	1	0.092	1.896	1	0.277	**3.804**	0.005	0.555
9	-2	0.927	-0.292	-0.367	1	-0.054	**3.265**	0.021	0.476
10	-0.109	1	-0.016	1.862	1	0.272	**3.301**	0.021	0.481
13	-2.54	0.29	-0.37	1.194	1	0.174	2.863	0.05	0.418
14	0.087	0.931	0.013	0.433	1	0.063	1.665	0.515	0.243
16	-2.801	0.164	-0.408	-0.173	1	-0.025	**3.009**	0.038	0.439
17	-2.659	0.225	-0.388	-0.016	0.987	-0.002	0.565	0.575	0.082
25	2.052	0.872	0.299	1.652	1	0.241	0.984	0.66	0.144
26	0.465	1	0.068	1.396	1	0.204	**4.395**	0.001	0.641
28	0.463	1	0.067	1.17	1	0.171	2.075	0.305	0.303
29	1.242	1	0.181	2.714	0.196	0.396	**4.808**	<0.001	0.701
30	-0.202	1	-0.029	0.158	1	0.023	**4.644**	<0.001	0.677
32	0.968	1	0.141	2.426	0.384	0.354	**4.185**	0.002	0.61
33	-0.554	1	-0.081	0.412	1	0.06	**4.449**	0.001	0.649
35	-0.257	1	-0.038	1.273	1	0.186	1.807	0.463	0.264
36	-0.359	1	-0.052	**3.267**	**0.045**	**0.477**	**5.09**	<0.001	0.742
39	-0.461	1	-0.067	1.794	1	0.262	1.415	0.656	0.206
40	-0.375	1	-0.055	1.316	1	0.192	**5.203**	<0.001	0.759

Bold indicates statistical significance.

Supplementary Table 4. One-sample t-test for deoxy-Hb

Verbal n-back task in younger adults (n = 49)

	0-back			1-back			2-back		
CH	t	P_{holm}	d	t	P_{holm}	d	t	P_{holm}	d
2	0.406	1	0.058	-0.016	1	-0.002	1.21	1	0.173
3	0.903	1	0.129	-0.397	1	-0.057	0.217	1	0.031
5	0.287	1	0.041	-1.131	1	-0.162	0.886	1	0.127
6	-0.013	0.989	-0.002	0.181	1	0.026	2.589	0.254	0.37
7	-1.509	1	-0.216	0.139	1	0.02	0.63	1	0.09
9	-1.108	1	-0.158	-0.014	1	-0.002	2.155	0.615	0.308
10	0.515	1	0.074	0.548	1	0.078	1.949	0.857	0.278
13	1.271	1	0.182	1.902	1	0.272	**3.494**	0.023	0.499
14	-1.626	1	-0.232	-0.075	1	-0.011	0.192	0.848	0.027
16	1.226	1	0.175	2.403	0.404	0.343	**3.316**	0.036	0.474
17	-0.407	1	-0.058	0.833	1	0.119	1.037	1	0.148
25	-0.054	1	-0.008	0.594	1	0.085	0.567	1	0.081
26	-0.597	1	-0.085	-0.284	1	-0.041	0.437	1	0.062
28	-2.004	1	-0.286	0.758	1	0.108	1.155	1	0.165
29	1.023	1	0.146	0.857	1	0.122	2.004	0.811	0.286
30	-0.411	1	-0.059	-0.155	1	-0.022	0.328	1	0.047
32	1.007	1	0.144	2.659	0.223	0.38	1.813	1	0.259
33	0.045	1	0.006	-0.107	1	-0.015	0.772	1	0.11
35	-0.724	1	-0.103	0.747	1	0.107	1.085	1	0.155
36	0.798	1	0.114	2.697	0.212	0.385	2.289	0.477	0.327
39	-0.148	1	-0.021	2.188	0.638	0.313	0.79	1	0.113
40	2.132	0.84	0.305	0.012	0.99	0.002	2.542	0.272	0.363

Spatial n-back task in younger adults (n = 49)

	0-back			1-back			2-back		
CH	t	P_{holm}	d	t	P_{holm}	d	t	P_{holm}	d
2	2.452	0.358	0.35	1.929	1	0.276	0.838	1	0.12
3	2.317	0.471	0.331	2.596	0.262	0.371	2.256	0.488	0.322
5	-0.153	1	-0.022	1.144	1	0.163	1.318	1	0.188
6	2.752	0.175	0.393	2.452	0.34	0.35	1.986	0.739	0.284
7	3.077	0.076	0.44	2.16	0.644	0.309	2.193	0.531	0.313
9	1.509	1	0.216	0.831	1	0.119	0.662	1	0.095
10	0.724	1	0.103	1.385	1	0.198	1.485	1	0.212
13	0.634	1	0.091	1.134	1	0.162	2.539	0.259	0.363
14	-0.423	1	-0.06	0.037	0.971	0.005	0.348	1	0.05
16	1.132	1	0.162	1.292	1	0.185	3.055	0.07	0.436
17	-0.018	0.986	-0.003	0.074	1	0.011	1.397	1	0.2
25	-0.425	1	-0.061	-1.232	1	-0.176	0.578	1	0.083
26	1.895	1	0.271	-1.831	1	-0.262	-0.036	0.971	-0.005
28	1.724	1	0.246	0.551	1	0.079	**3.67**	0.013	0.524
29	0.642	1	0.092	1.439	1	0.206	0.102	1	0.015
30	2.045	0.835	0.292	0.643	1	0.092	1.261	1	0.18
32	0.642	1	0.092	2.717	0.201	0.388	2.093	0.624	0.299
33	1.218	1	0.174	1.801	1	0.257	0.531	1	0.076
35	-0.701	1	-0.1	0.875	1	0.125	1.283	1	0.183
36	1.403	1	0.2	2.53	0.294	0.361	3.179	0.052	0.454
39	-1.14	1	-0.163	1.217	1	0.174	0.756	1	0.108
40	0.972	1	0.139	1.911	0.992	0.273	**4.205**	0.002	0.601

Bold indicates statistical significance.

(continued)

Verbal n-back task in older adults (n = 47)

CH	0-back			1-back			2-back		
	t	P_{holm}	d	t	P_{holm}	d	t	P_{holm}	d
2	2.18	0.619	0.318	1.074	1	0.157	1.52	0.945	0.222
3	**3.764**	**0.01**	**0.549**	2.659	0.214	0.388	1.565	1	0.228
5	0.424	1	0.062	-0.289	1	-0.042	1.357	0.905	0.198
6	0.845	1	0.123	1.742	1	0.254	2.444	0.239	0.357
7	0.823	1	0.12	-0.834	1	-0.122	1.819	0.755	0.265
9	2.8	0.156	0.408	0.926	1	0.135	**4.091**	0.003	0.597
10	-1.702	1	-0.248	0.723	1	0.105	1.585	1	0.231
13	-0.381	1	-0.056	-0.544	1	-0.079	1.25	0.872	0.182
14	-1.812	1	-0.264	-0.073	0.942	-0.011	0.76	0.902	0.111
16	-0.898	1	-0.131	-0.194	1	-0.028	1.928	0.661	0.281
17	-1.579	1	-0.23	-0.748	1	-0.109	-1.066	0.876	-0.156
25	2.538	0.292	0.37	1.45	1	0.212	2.909	0.089	0.424
26	0.199	1	0.029	1.318	1	0.192	1.47	0.888	0.214
28	0.85	1	0.124	1.524	1	0.222	**4.355**	0.002	0.635
29	0.38	1	0.055	2.71	0.198	0.395	**3.329**	0.033	0.486
30	0.91	1	0.133	-0.147	1	-0.021	2.414	0.238	0.352
32	0.159	0.875	0.023	3.085	0.076	0.45	**3.269**	0.037	0.477
33	2.473	0.325	0.361	2.583	0.247	0.377	**4.458**	0.001	0.65
35	-1.827	1	-0.267	-0.565	1	-0.082	0.643	0.524	0.094
36	-0.2	1	-0.029	1.886	1	0.275	2.853	0.097	0.416
39	-1.721	1	-0.251	1.035	1	0.151	2.631	0.161	0.384
40	0.544	1	0.079	2.358	0.409	0.344	**3.187**	0.044	0.465

Spatial n-back task in older adults (n = 47)

CH	0-back			1-back			2-back		
	t	P_{holm}	d	t	P_{holm}	d	t	P_{holm}	d
2	0.996	1	0.145	0.221	1	0.032	2.615	0.132	0.381
3	-0.873	1	-0.127	-0.715	1	-0.104	2.869	0.081	0.418
5	0.649	1	0.095	0.766	1	0.112	2.913	0.077	0.425
6	-1.343	1	-0.196	0.483	1	0.07	**3.728**	0.01	0.544
7	-1.179	1	-0.172	0.229	1	0.033	1.466	0.75	0.214
9	0.965	1	0.141	**3.642**	**0.014**	**0.531**	**4.602**	0.001	0.671
10	-0.574	1	-0.084	**3.48**	**0.022**	**0.508**	2.166	0.285	0.316
13	-2.001	1	-0.292	**3.656**	**0.015**	**0.533**	1.846	0.428	0.269
14	-0.975	1	-0.142	0.098	0.923	0.014	-0.111	1	-0.016
16	-0.762	1	-0.111	**3.263**	**0.04**	**0.476**	**3.609**	0.011	0.526
17	-1.987	1	-0.29	1.497	1	0.218	-0.495	1	-0.072
25	-0.956	1	-0.139	0.928	1	0.135	**3.627**	0.012	0.529
26	0.594	1	0.087	-0.593	1	-0.087	1.981	0.375	0.289
28	1.222	1	0.178	1.858	1	0.271	**3.62**	0.012	0.528
29	-0.722	1	-0.105	1.27	1	0.185	**3.713**	0.01	0.542
30	0.445	1	0.065	0.418	1	0.061	2.664	0.127	0.389
32	1.437	1	0.21	2.102	0.697	0.307	**4.48**	0.001	0.654
33	-0.983	1	-0.143	2.517	0.277	0.367	**4.268**	0.002	0.623
35	0.159	0.875	0.023	-0.266	1	-0.039	0.042	0.966	0.006
36	-0.995	1	-0.145	1.814	1	0.265	2.481	0.151	0.362
39	-1.42	1	-0.207	1.353	1	0.197	0.359	1	0.052
40	-0.911	1	-0.133	2.046	0.744	0.298	2.594	0.127	0.378

Bold indicates statistical significance.

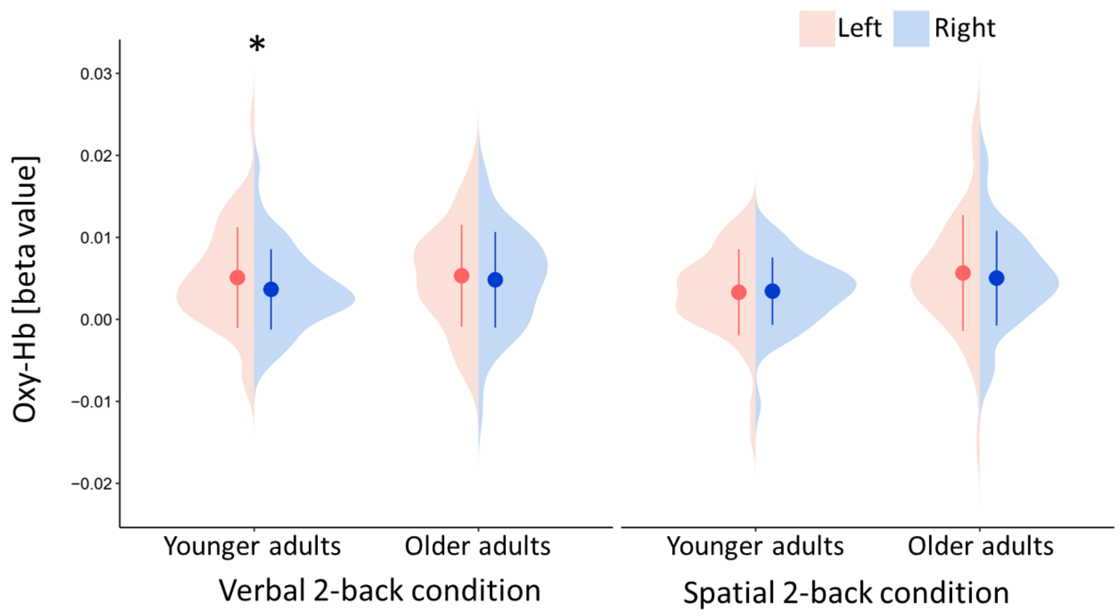

Supplementary Figure 7. Oxy-Hb activation patterns of the left and right hemispheres for verbal and spatial 2-back conditions. Only channels with significant activation were averaged by hemisphere. Half-violin plots show the brain activation distribution for both hemispheres (red: left side, blue: right side). Circles and error bars indicate mean and standard error, respectively. *$p < 0.05$

Supplementary Table 5. Results of two-way ANCOVA for oxy-Hb of each CH during each n-back task

Oxy-Hb

		Main effect						Interaction		
		Group			Memory load			Group × Memory load		
Task	CH	F	P_{holm}	η_p^2	F	P_{holm}	η_p^2	F	P_{holm}	η_p^2
Verbal n-back	2	0.84	1	0.009	1.95	1	0.02	2.42	1	0.03
	3	0.01	0.92	<0.001	1.58	1	0.02	3.23	0.775	0.03
	5	5.84	0.42	0.06	0.29	1	0.003	1.42	1	0.02
	6	0.11	1	<0.001	0.14	1	0.002	1.88	1	0.02
	7	2.42	1	0.03	0.08	1	<0.001	**10.81**	0.002	0.11
	9	0.12	1	<0.001	0.07	1	<0.001	2.09	1	0.02
	10	0.09	1	<0.001	1.18	1	0.01	5.75	0.12	0.06
	13	2.09	1	0.02	0.15	1	0.002	0.28	1	0.003
	14	1.59	1	0.02	0.47	1	0.005	5.05	0.148	0.05
	16	0.12	1	<0.001	0.49	1	0.005	1.18	1	0.01
	17	2.22	1	0.02	0.78	1	0.008	1.17	1	0.01
	25	2.96	1	0.03	0.45	1	0.005	3.12	0.775	0.03
	26	7.66	0.154	0.08	2.47	1	0.03	3.58	0.51	0.04
	28	0.59	1	0.006	0.11	1	<0.001	**8.98**	0.011	0.09
	29	4.94	0.6	0.05	0.52	1	0.006	5.07	0.148	0.05
	30	3.44	1	0.04	0.34	1	0.004	0.44	1	0.005
	32	0.07	1	<0.001	0.01	0.99	<0.001	2.8	0.98	0.03
	33	0.91	1	0.01	0.1	1	<0.001	1.79	1	0.02
	35	0.07	1	<0.001	0.21	1	0.002	1.4	1	0.01
	36	1.09	1	0.01	0.45	1	0.005	1.59	1	0.02
	39	0.2	1	0.002	0.03	1	<0.001	0.25	0.77	0.003
	40	0.72	1	0.008	0.78	1	0.008	0.46	1	0.005
Spatial n-back	2	2.32	1	0.02	1.37	1	0.01	2.57	1	0.03
	3	2.15	1	0.02	3.57	0.84	0.04	1.28	1	0.01
	5	1.85	1	0.02	0.29	1	0.003	5.86	0.105	0.06
	6	0.24	1	0.003	1.53	1	0.02	2.98	0.9	0.03
	7	2.65	1	0.03	1.33	1	0.01	4.25	0.39	0.04
	9	0.39	1	0.004	0.46	1	0.005	3.12	0.8	0.03
	10	0.07	1	<0.001	1.6	1	0.02	1.66	1	0.02
	13	2.9	1	0.03	0.31	1	0.003	0.82	1	0.009
	14	1.01	1	0.01	0.1	1	<0.001	0.64	1	0.007
	16	1.27	1	0.01	0.54	1	0.006	0.25	1	0.003
	17	5.43	0.44	0.06	0.8	1	0.009	0.05	0.94	<0.001
	25	0.01	0.93	<0.001	1.36	1	0.01	0.61	1	0.007
	26	0.33	1	0.004	0.1	1	<0.001	4.19	0.39	0.04
	28	0.04	1	<0.001	0.24	1	0.003	1.41	1	0.02
	29	0.08	1	<0.001	0.26	1	0.003	3.7	0.525	0.04
	30	0.72	1	0.008	0.1	1	<0.001	3.51	0.525	0.04
	32	0.13	1	<0.001	0.79	1	0.009	1.39	1	0.01
	33	0.27	1	0.003	0.39	1	0.004	**6.51**	0.044	0.07
	35	0.49	1	0.005	1.54	1	0.02	0.76	1	0.008
	36	0.17	1	0.002	0.19	1	0.002	0.18	1	0.002
	39	1.55	1	0.02	3.58	0.66	0.04	1.58	1	0.02
	40	0.01	1	<0.001	0.01	0.99	<0.001	0.29	1	0.003

Bold indicates statistical significance.

(continued)

Deoxy-Hb

		Main effect						Interaction		
		Group			Memory load			Group × Memory load		
Task	CH	F	P_{holm}	η_p^2	F	P_{holm}	η_p^2	F	P_{holm}	η_p^2
Verbal n-back	2	4.56	0.76	0.05	1.82	1	0.02	0.5	1	0.005
	3	5.69	0.44	0.06	3.05	1	0.03	2.33	1	0.02
	5	2.1	1	0.02	1.68	1	0.02	0.69	1	0.007
	6	1.65	1	0.02	1.04	1	0.01	0.32	1	0.003
	7	3.21	1	0.03	0.77	1	0.008	**1.36**	**1**	**0.01**
	9	4.64	0.615	0.05	1.31	1	0.01	1.64	1	0.02
	10	0.21	1	0.002	1.27	1	0.01	0.37	1	0.004
	13	1.71	1	0.02	0.41	1	0.004	0.58	1	0.006
	14	0.47	1	0.005	0.96	1	0.01	0.25	1	0.003
	16	1.03	1	0.01	1.07	1	0.01	0.36	1	0.004
	17	0.59	1	0.006	1.35	1	0.01	0.61	1	0.007
	25	3.1	1	0.03	0.07	0.93	<0.001	1.24	1	0.01
	26	1.07	1	0.01	1.22	1	0.01	0.13	0.86	<0.001
	28	5.05	0.615	0.05	2.01	1	0.02	**2.27**	**1**	**0.02**
	29	0.81	1	0.009	0.5	1	0.005	1.33	1	0.01
	30	1.17	1	0.01	0.13	1	<0.001	0.2	1	0.002
	32	0.02	0.89	<0.001	0.34	1	0.004	1.6	1	0.02
	33	4	0.9	0.04	1.12	1	0.01	1.52	1	0.02
	35	0.32	1	0.003	1.61	1	0.02	0.56	1	0.006
	36	0.04	1	<0.001	1.6	1	0.02	1.97	1	0.02
	39	0.02	1	<0.001	0.09	1	<0.001	1.84	1	0.02
	40	1.54	1	0.02	3.31	0.88	0.03	3.65	0.66	0.04
Spatial n-back	2	0.08	1	<0.001	0.5	1	0.005	3.13	1	0.03
	3	3.53	1	0.04	1.81	1	0.02	1.93	1	0.02
	5	0.33	1	0.004	0.02	0.97	<0.001	1.06	1	0.01
	6	0.3	1	0.003	0.32	1	0.003	6.47	0.082	0.07
	7	4.44	0.86	0.05	0.51	1	0.005	1.73	1	0.02
	9	4.31	0.86	0.04	1.54	1	0.02	6.21	0.082	0.06
	10	0.15	1	0.002	0.35	1	0.004	0.32	1	0.003
	13	0.64	1	0.007	0.84	1	0.009	0.87	1	0.009
	14	0.02	1	<0.001	0.97	1	0.01	0.21	0.78	0.002
	16	0.2	1	0.002	1.34	1	0.01	2.55	1	0.03
	17	0.38	1	0.004	2.33	1	0.02	0.42	1	0.005
	25	1.46	1	0.02	2.66	1	0.03	2.21	1	0.02
	26	0.47	1	0.005	0.8	1	0.009	1.74	1	0.02
	28	0	0.98	<0.001	0.17	1	0.002	0.71	1	0.008
	29	0.84	1	0.009	2.58	1	0.03	7.75	0.015	0.08
	30	0.11	1	<0.001	1.85	1	0.02	1.08	1	0.01
	32	0.09	1	<0.001	0.82	1	0.009	2.69	1	0.03
	33	0.26	1	0.003	0.93	1	0.01	**5.28**	0.133	0.05
	35	0.03	1	<0.001	0.43	1	0.005	0.33	1	0.004
	36	2.05	1	0.02	1.39	1	0.01	0.3	1	0.003
	39	0.2	1	0.002	3.53	0.88	0.04	0.98	1	0.01
	40	0.28	1	0.003	0.44	1	0.005	0.77	1	0.008

Bold indicates statistical significance.

Supplementary Table 6. Post-hoc comparisons after ANCOVA for oxy- and deoxy-Hb activation

Simple main effect of memory load (0-back/1-back/2-back)

				0-back vs 1-back			0-back vs 2-back			1-back vs 2-back		
Group	Task	Oxy/Deoxy	CH	t	P_{holm}	d	t	P_{holm}	d	t	P_{holm}	d
Younger adults	Verbal	Oxy-Hb	7	1.507	0.135	0.222	1.295	0.199	0.184	0.274	0.785	0.044
			28	-0.465	0.643	-0.063	0.280	0.781	0.041	0.652	0.516	0.095
	Spatial	Oxy-Hb	33	**-4.186**	0.000	-0.614	**-2.889**	0.005	-0.416	0.948	0.346	0.131
		Deoxy-Hb	29	-0.831	0.408	-0.119	0.981	0.329	0.143	1.670	0.098	0.245
Older adults	Verbal	Oxy-Hb	7	-0.502	0.617	-0.073	**-4.540**	0.000	-0.667	**-4.497**	0.000	-0.662
			28	-0.671	0.504	-0.097	**-4.460**	0.000	-0.667	**-4.506**	0.000	-0.640
	Spatial	Oxy-Hb	33	-0.297	0.767	-0.040	**-4.561**	0.000	-0.690	**-3.923**	0.000	-0.583
		Deoxy-Hb	29	-1.233	0.221	-0.194	**-3.993**	0.000	-0.625	**-2.955**	0.004	-0.458

Simple main effect of group (younger adults vs older adults)

			0-back			1-back			2-back		
Task	Oxy/Deoxy	CH	t	P_{holm}	d	t	P_{holm}	d	t	P_{holm}	d
Verbal	Oxy-Hb	7	1.082	0.282	0.219	-0.288	0.774	-0.064	**-3.535**	0.001	-0.720
		28	0.989	0.325	0.204	0.806	0.422	0.175	**-2.697**	0.008	-0.551
Spatial	Oxy-Hb	33	-1.095	0.277	-0.223	1.980	0.051	0.408	-1.961	0.053	-0.408
	Deoxy-Hb	29	1.099	0.275	0.233	0.639	0.525	0.128	**-2.788**	0.006	-0.538

Bold indicates statistical significance.

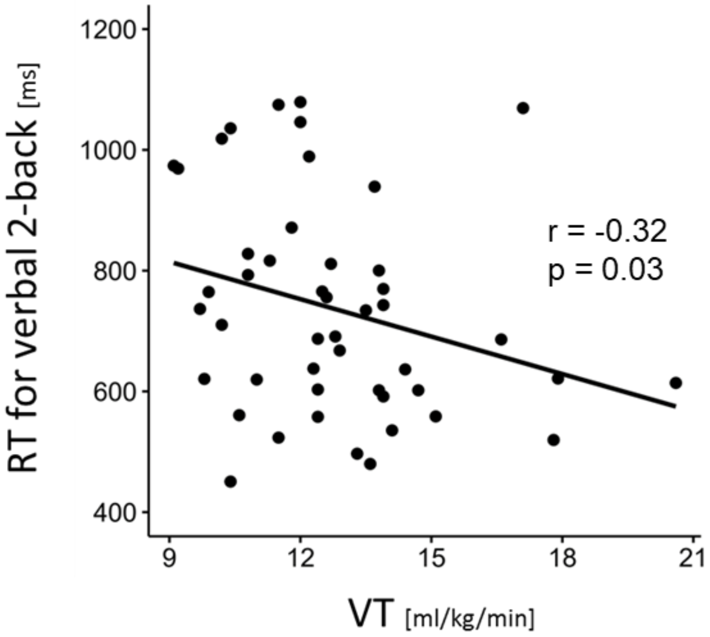

Supplementary Figure 8. Scatter plot between ventilatory threshold (VT) and reaction time (RT) for verbal 2-back condition in older adults (n = 47)

Supplementary Table 7. Correlations of oxy- and deoxy-Hb activation in verbal 2-back condition with VT and RT

	VT		RT	
CH	r_p	p	r_p	p
Oxy-Hb				
2	0.266	0.081	-0.101	0.515
3	0.155	0.315	0.04	0.796
5	0.087	0.576	-0.17	0.271
6	0.164	0.287	-0.09	0.56
7	**0.368**	0.014	**-0.334**	0.026
9	0.268	0.078	-0.279	0.067
10	**0.339**	0.024	-0.206	0.18
13	0.248	0.105	**-0.315**	0.038
14	**0.356**	0.018	-0.266	0.081
16	0.136	0.38	**-0.305**	0.044
17	**0.328**	0.03	**-0.31**	0.041
25	0.23	0.134	-0.082	0.595
26	0.241	0.115	**-0.315**	0.038
28	**0.408**	0.006	**-0.342**	0.023
29	**0.39**	0.009	**-0.353**	0.019
30	0.08	0.604	-0.166	0.28
32	0.152	0.325	-0.081	0.602
33	0.132	0.395	-0.17	0.269
35	0.276	0.069	-0.112	0.47
36	**0.387**	0.009	-0.217	0.157
39	**0.363**	0.015	**-0.258**	0.09
40	0.275	0.071	-0.086	0.577
Deoxy-Hb				
2	0.047	0.764	-0.298	0.05
3	0.153	0.321	**-0.356**	0.018
5	-0.073	0.637	0.091	0.555
6	0.19	0.216	-0.215	0.162
7	0.13	0.4	0.03	0.848
9	0.062	0.689	0.061	0.695
10	**0.312**	0.039	-0.278	0.067
13	0.031	0.839	**-0.299**	0.049
14	**0.31**	0.04	-0.088	0.57
16	0.004	0.981	-0.052	0.737
17	0.073	0.64	-0.328	0.03
25	0.161	0.295	-0.22	0.152
26	0.007	0.966	0.15	0.33
28	0.209	0.173	-0.064	0.681
29	0.092	0.555	0.082	0.597
30	0.171	0.266	0.034	0.825
32	0.144	0.351	-0.195	0.205
33	0.236	0.124	-0.127	0.411
35	0.158	0.305	0.012	0.939
36	0.108	0.486	-0.13	0.4
39	0.238	0.119	-0.179	0.244
40	0.092	0.554	-0.008	0.958

Correlation coefficients were calculated using partial Pearson correlation analysis controlling for age, sex, and years of education. Bold indicates statistical significance.
